# An archaeal ADP-dependent serine kinase involved in cysteine biosynthesis and serine metabolism

**DOI:** 10.1038/ncomms13446

**Published:** 2016-11-18

**Authors:** Yuki Makino, Takaaki Sato, Hiroki Kawamura, Shin-ichi Hachisuka, Ryo Takeno, Tadayuki Imanaka, Haruyuki Atomi

**Affiliations:** 1 Department of Synthetic Chemistry and Biological Chemistry, Graduate School of Engineering, Kyoto University, Katsura, Nishikyo-ku, Kyoto 615-8510, Japan; 2 JST, CREST, 7, Gobancho, Chiyoda-ku, Tokyo 102-0076, Japan; 3 Research Organization of Science and Technology, Ritsumeikan University, Noji-Higashi, Kusatsu 525-8577, Japan

## Abstract

Routes for cysteine biosynthesis are still unknown in many archaea. Here we find that the hyperthermophilic archaeon *Thermococcus kodakarensis* generates cysteine from serine via *O*-phosphoserine, in addition to the classical route from 3-phosphoglycerate. The protein responsible for serine phosphorylation is encoded by TK0378, annotated as a chromosome partitioning protein ParB. The TK0378 protein utilizes ADP as the phosphate donor, but in contrast to previously reported ADP-dependent kinases, recognizes a non-sugar substrate. Activity is specific towards free serine, and not observed with threonine, homoserine and serine residues within a peptide. Genetic analyses suggest that TK0378 is involved in serine assimilation and clearly responsible for cysteine biosynthesis from serine. TK0378 homologs, present in Thermococcales and Desulfurococcales, are most likely not ParB proteins and constitute a group of kinases involved in serine utilization.

## Abstract (web summary)

Archaea metabolism has unique adaptations to hostile environments. Here Makino *et al*. describe an unusual ADP-dependent kinase that phosphorylates free serine to *O*-phosphoserine and participates in an additional cysteine biosynthetic pathway in the archaeon *Thermococcus kodakarensis*.

## Graphical abstract

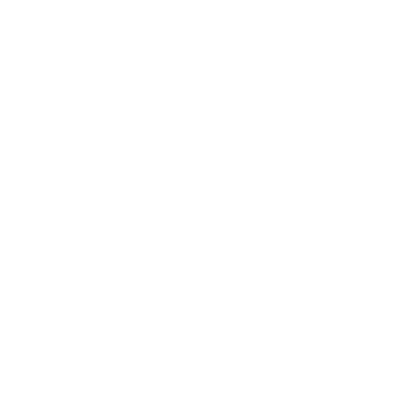

Members of the Archaea, which form the third domain of life, exhibit metabolic pathways that are distinct to those found in bacteria and eukaryotes. In case of central carbon metabolism, modified versions of the Embden-Meyerhof and Entner-Doudoroff pathways have been reported^1^,^2^,^3^,^4^. In terms of biosynthesis, distinct pathways to generate pentoses^5^,^6^, coenzyme A^7^ and polyamines^8^,^9^ have been identified. In addition, several carbon dioxide fixing pathways^2^,^10^,^11^,^12^ and a nucleotide/nucleoside degradation pathway^13^,^14^ have been discovered in Archaea.

L-Cysteine (Cys) is an important amino acid that provides thiol groups in both proteins and other biomolecules such as coenzyme A. The pathway for Cys biosynthesis is still unknown in many archaeal species. Until now, four pathways/mechanisms have been identified for Cys biosynthesis in bacteria, eukaryotes and several archaeal species. One, found mainly in mammals and yeast, initiates from Met and leads to Cys via *S*-adenosylmethionine, *S*-adenosylhomocysteine, homocysteine and cystathionine (Fig. 1a, pathway Cys1) (ref. 15). In this pathway, cystathionine γ-lyase (CGL) catalyses the cleavage of cystathionine to generate Cys and 2-oxobutyrate. Another pathway generates Cys from 3-phosphoglycerate via 3-phosphohydroxypyruvate and *O*-phosphoserine (Sep) (pathway Cys2), which was identified in the hyperthermophilic archaeon *Aeropyrum pernix*^16^. In this pathway, 3-phosphoglycerate dehydrogenase catalyses the conversion of 3-phosphoglycerate to 3-phosphohydroxypyruvate. The third pathway is predominantly found in bacteria and plants and generates Cys from L-serine (Ser) via *O*-acetylserine (pathway Cys3) (refs 17, 18). Serine acetyltransferase is a key enzyme in this pathway. In addition, there is a unique mechanism, discovered in methanogenic archaea and *Archaeoglobus*, in which Cys is generated as cysteinyl transfer RNA (tRNA)^Cys^ for its use in protein synthesis^19^,^20^. In this case, tRNA^Cys^ is first aminoacylated with Sep to form phosphoseryl tRNA^Cys^ (Sep-tRNA^Cys^), and the Sep to Cys conversion occurs in a tRNA-linked manner. These mechanisms for Cys biosynthesis, including the tRNA-dependent mechanism, are summarized in Supplementary Fig. 1.

*Thermococcus kodakarensis* is a hyperthermophilic archaeon isolated from Kodakara Island, Japan^21^. The entire genome has been sequenced^22^, and several genetic systems have been developed^23^,^24^,^25^, allowing the deletion and introduction of genes in this archaeon. In this study, we aimed to elucidate the pathways responsible for Cys biosynthesis in *T. kodakarensis*.

Here we find that *T. kodakarensis* generates Cys from Ser via Sep, in addition to the classical route from 3-phosphoglycerate. Furthermore, Ser is found to be produced from L-threonine (Thr) via L-glycine (Gly) as well as from 3-phosphoglycerate via Sep. In Cys biosynthesis, the protein responsible for Ser phosphorylation is encoded by TK0378, annotated as a chromosome partitioning protein ParB. This protein utilizes ADP as the phosphate donor, being a kinase that recognizes free Ser. Genetic analyses suggest that TK0378 is involved in Ser assimilation and clearly responsible for Cys biosynthesis from Ser.

## Results

### Cys biosynthesis in *T. kodakarensis*

The hyperthermophilic archaeon *T. kodakarensis* can grow in a defined synthetic medium composed only of 20 amino acids as the main carbon source (artificial sea water (ASW)-AA-S^0^). We found that the organism can also grow when Cys is excluded from this medium (see below), indicating that *T. kodakarensis* can generate Cys from the other 19 amino acids. However, the pathway(s) responsible for Cys biosynthesis could not be clearly identified based on the genome sequence alone^22^.

### Evaluation of established Cys biosynthesis pathways

**Figure 1:**
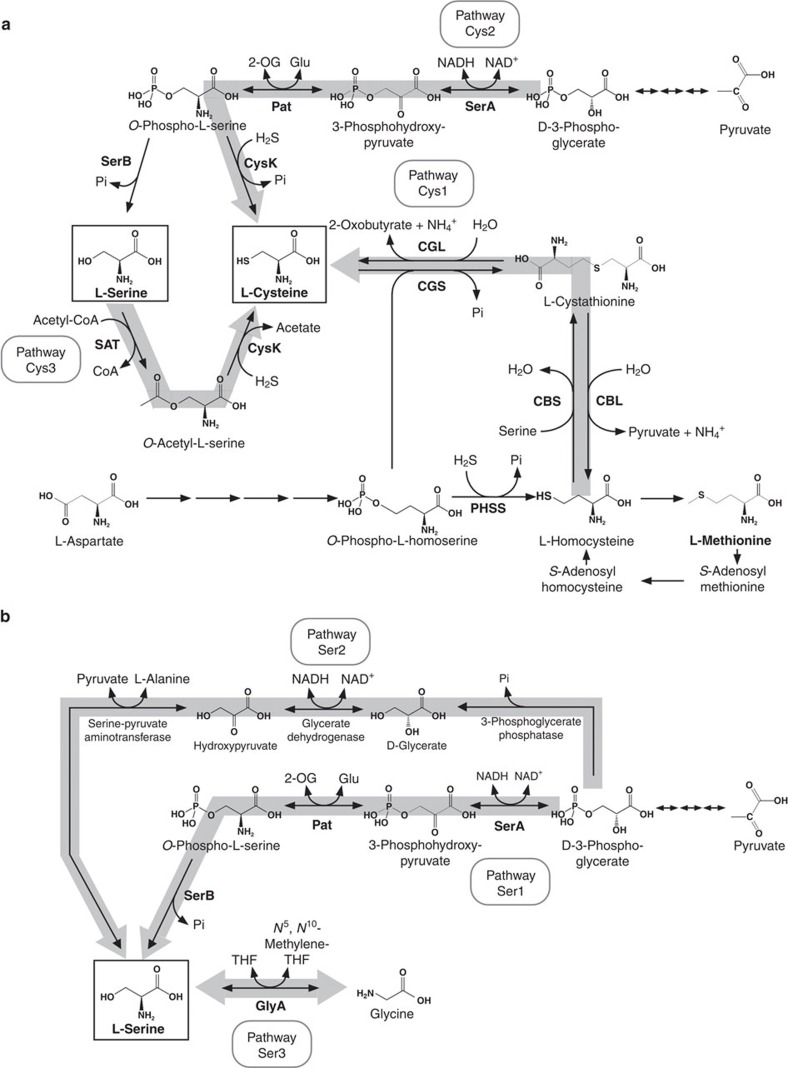
The three previously known pathways for Cys and Ser biosyntheses. (**a**) The three pathways for Cys biosynthesis (Pathway Cys1, Cys2 and Cys3) are highlighted with grey arrows. In some bacteria, *O*-phosphohomoserine is replaced by *O*-acetylhomoserine. (**b**) The three previously known pathways for Ser biosynthesis (Pathway Ser1, Ser2 and Ser3) are highlighted with grey arrows. Compound abbreviations: 2-OG, 2-oxoglutarate, Pi, phosphate; THF, tetrahydrofolate. Enzyme abbreviations: CBL, cystathionine β-lyase; CBS, cystathionine β-synthase; CGL, cystathionine γ-lyase; CGS, cystathionine γ-synthase; CysK, cysteine synthase; GlyA, glycine/serine hydroxymethyltransferase; Pat, phosphoserine aminotransferase; PHSS, phosphohomoserine sulfhydrylase; SAT, serine acetyltransferase.

**Figure 2:**
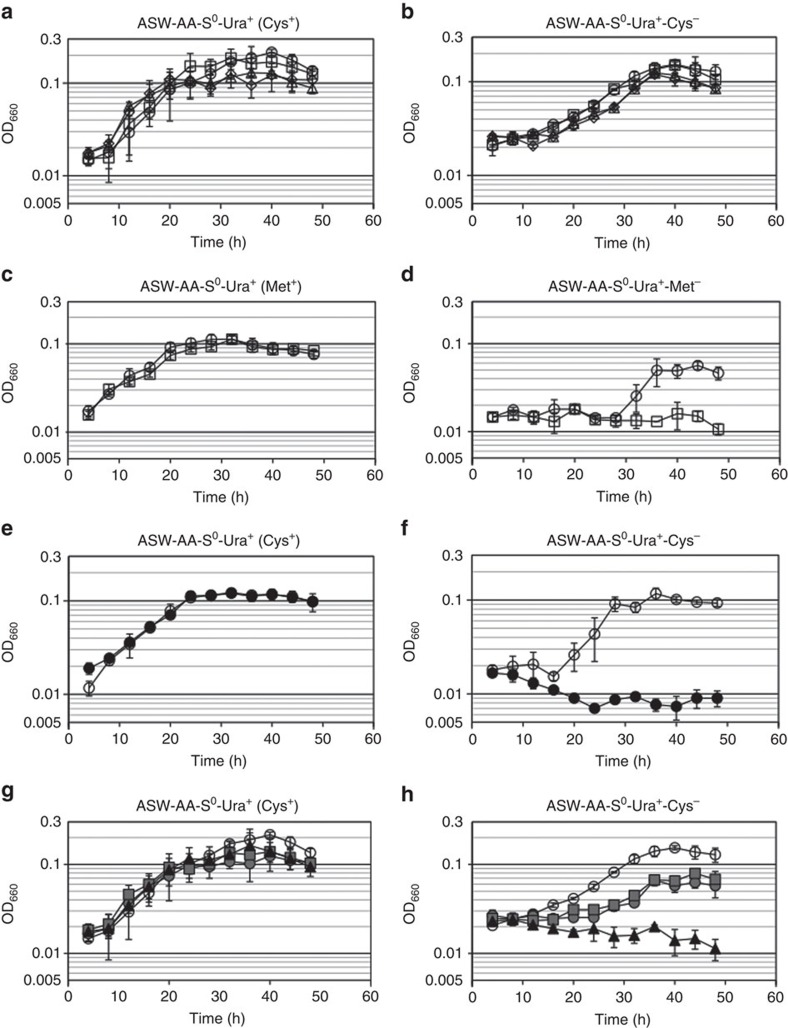
Cys or Met auxotrophy of *Thermococcus kodakarensis* gene disruptants. (**a**,**b**,**e**–**h**) Cys auxotrophy was investigated by cultivating cells in ASW-AA-S^0^-Ura^+^ medium with (**a**,**e**,**g**) or without Cys (**b**,**f**,**h**). Symbols: *T. kodakarensis* KU216 (open circles), Δ*tk1449*-2-1-2 (open squares), Δ*serA*Δ*ldhA1*Δ*ldhA2*-1 (open triangles), Δ*tk1449*Δ*serA*Δ*ldhA1*Δ*ldhA2*-2 (open diamonds), Δ*cysK*-3 (black circles), Δ*serK*-1 (grey circles), Δ*tk1449*Δ*serK*-1 (grey squares) and Δ*serA*Δ*ldhA1*Δ*ldhA2*Δ*serK*-1 (black triangles). (**c**,**d**) Met auxotrophy was examined by cultivating cells in ASW-AA-S^0^-Ura^+^ medium with (**c**) or without Met (**d**). Symbols: *T. kodakarensis* KU216 (open circles) and Δ*tk1449*-2-1-2 (open squares). Error bars indicate the standard deviations of three independent culture experiments.

In order to identify the pathway(s) involved in Cys biosynthesis in *T. kodakarensis*, we disrupted genes presumed to encode the enzymes of each previously identified pathway. After isolating the transformants, genotypes were confirmed by PCR (Supplementary Figs 2 and 3, Supplementary Table 1). We first examined the involvement of pathway Cys1 (Fig. 1a), which consists of cystathionine β-synthase (CBS) and CGL. There was not a very strong candidate for a CBS in *T. kodakarensis*. The TK1687 protein is 34% identical to the N-terminal region of the CBS from *Saccharomyces cerevisiae* S288c (507 residues), but is much smaller in size (273 residues). On the other hand, the TK1687 protein is similar in both size and primary structure to the archaeal cysteine synthases (CysK) from *Methanosarcina thermophila* (37% identical) and *A. pernix* (36%). Concerning CGL, the TK1449 protein product is 38% identical to the CGL from *S. cerevisiae* S288c. Other genes with notable similarity to CGL were not found on the genome. In order to examine the possibility that TK1449 encodes a CGL and that Cys is synthesized via pathway Cys1, we constructed a TK1449 gene disruption strain. Growth of the Δ*tk1449* mutant in a synthetic medium without Cys was similar to that of the host strain (Fig. 2a,b), suggesting that the main pathway for Cys biosynthesis is not pathway Cys1. On the other hand, the TK1449 protein is also 25% identical to cystathionine β-lyase (CBL) and 35% identical to cystathionine γ-synthase (CGS) from *E. coli*, which function for Met biosynthesis. When we examined the effects of TK1449 disruption on Met biosynthesis in *T. kodakarensis*, we observed clear Met auxotrophy (Fig. 2c,d), suggesting that this gene is involved in Met biosynthesis, most likely encoding a CGS and/or a CBL, involved in the conversion of *O*-phosphohomoserine to L-homocysteine via L-cystathionine (Fig. 1a). The TK1449 protein may also function as a phosphohomoserine sulfhydrylase, an activity detected in plants that directly converts *O*-phosphohomoserine to L-homocysteine (Fig. 1a).

Next, we evaluated the involvement of pathway Cys2, through which Cys is synthesized from 3-phosphoglycerate via Sep. A gene predicted to encode 3-phosphoglycerate dehydrogenase (SerA) is present on the genome (*Tk-serA*: TK1966 (42% identical to SerA from *E. coli*)). We thus attempted to inactivate this pathway by disrupting the *serA* gene. As two other homologs with relatively high similarity, annotated as lactate dehydrogenases (LDHs), were also present (*Tk-ldhA1*: TK0551 (29% identical), *Tk-ldhA2*: TK0683 (31% identical)), we constructed a triple gene disruption strain of these genes to ensure the complete shutdown of pathway Cys2. As a result, growth without Cys was not significantly affected by the *serA*-*ldhA1*-*ldhA2* triple gene disruption (Fig. 2a,b), indicating that the main route for Cys biosynthesis is not pathway Cys2 under these conditions.

To exclude the possibilities that pathways Cys1 and Cys2 each contribute partially to Cys biosynthesis, we constructed a strain disrupted of both pathways Cys1 and Cys2 (Δ*tk1449* Δ*serA* Δ*ldhA1* Δ*ldhA2*). As shown in Fig. 2a,b, growth of the disruption strain was similar to that of the host strain in the absence of Cys, suggesting that pathways starting from Ser such as pathway Cys3, are responsible for Cys biosynthesis.

### The possibility of Cys biosynthesis via *O*-phosphoserine

Concerning pathway Cys3 (Fig. 1a), we could not identify a clear serine acetyltransferase homolog on the *T. kodakarensis* genome. On the other hand, as described above, a homolog of the other enzyme in this pathway, CysK, was present (*Tk-cysK*: TK1687). We confirmed that the *Tk-cysK* gene actually contributes in Cys biosynthesis. A *Tk-cysK* gene disruptant clearly displayed Cys auxotrophy as shown in Fig. 2e,f. The primary structure of *Tk-*CysK was then examined in detail. *Tk*-CysK possesses insertion sequences found in CysKs utilizing Sep rather than *O*-acetylserine (Supplementary Fig. 4) (ref. 26). We also found the positively-charged residue Lys^188^ corresponding to Arg^297^ involved in Sep recognition in the CysK from *A. pernix*^27^. Although Sep is generally synthesized from 3-phosphoglycerate, gene disruptions blocking pathway Cys2 did not lead to Cys auxotrophy (Fig. 2a,b), as described above. The *Tk-*CysK structure and our results raised the possibility that Ser is directly phosphorylated to Sep, which is subsequently converted to Cys by *Tk-*CysK.

### An ADP-dependent Ser kinase activity in *Thermococcus*

To our knowledge, although protein kinases that act on Ser residues in proteins are abundant, a kinase phosphorylating free Ser is not known. Nevertheless, we examined ATP-dependent Ser kinase activity in *T. kodakarensis* cell-free extracts (CFEs). The ADP produced from ATP in the kinase reaction was quantified with coupling enzymes. However, Ser-dependent generation of ADP was not detected. On the contrary, we noticed that the concentration of ADP in the assay mixture after the reaction with Ser (0.25±0.01 mM,±represents the standard deviation of three independent experiments) was actually smaller than that without Ser (0.45±0.01 mM). This suggested the presence of a Ser-dependent reaction that consumes ADP. ADP is present at trace levels in commercially available ATP and is also generated by thermal degradation of ATP during the reaction. Taking into account that several archaeal species utilize ADP-dependent sugar kinases (glucokinase^28^, phosphofructokinase^29^ in *Pyrococcus*, *Thermococcus*, *Archaeoglobus* and some methanogens (including *Methanococcales* and *Methanosarcinales*) and ribose-1-phosphate kinase in *Thermococcus* and *Pyrococcus*^14^), we considered the possibility that *T. kodakarensis* harbours an ADP-dependent Ser kinase. We thus examined Ser-dependent AMP generation from ADP in CFEs using high-performance liquid chromatography (HPLC). As a result, AMP was formed from ADP only in the presence of Ser (Supplementary Fig. 5), suggesting the occurrence of an ADP-dependent Ser kinase in *T. kodakarensis*.

### Identification and examination of ADP-dependent Ser kinase

In order to identify the ADP-dependent Ser kinase, proteins with Ser-dependent AMP generating activity from ADP were purified from *T. kodakarensis* CFEs. After each purification step, the AMP forming activities in each fraction were measured by HPLC and fractions with high activity were chosen and further purified as described in the Methods section. As a result, three candidate proteins, whose concentrations seemed to correlate with the ADP-dependent Ser kinase activity, were identified (Supplementary Fig. 6). Liquid chromatography–mass spectrometry (LC-MS) analysis revealed that the three candidates were the products of TK0378, TK1110 and TK1998 annotated as chromosome partitioning protein ParB, ADP-dependent glucokinase and hypothetical protein, respectively (Supplementary Table 2). ParB is a protein present in a wide range of bacteria and is involved in a variety of functions including chromosome partitioning and sporulation regulation^30^. A ParB encoded in the plasmid RK2, which is maintained in a broad range of Gram-negative bacteria, exhibits nuclease activity^31^. A closely related TK0378 homolog from *Pyrococcus furiosus* (PF0380) has been studied and also reported to exhibit nuclease activity^32^. The TK1110 homolog from *P. furiosus* is an ADP-dependent glucokinase^28^, responsible for the first step in glycolysis. Taking into account that the *P. furiosus* glucokinase is ADP-dependent, we presumed that TK1110 might recognize dual substrates, glucose and Ser.

**Figure 3:**
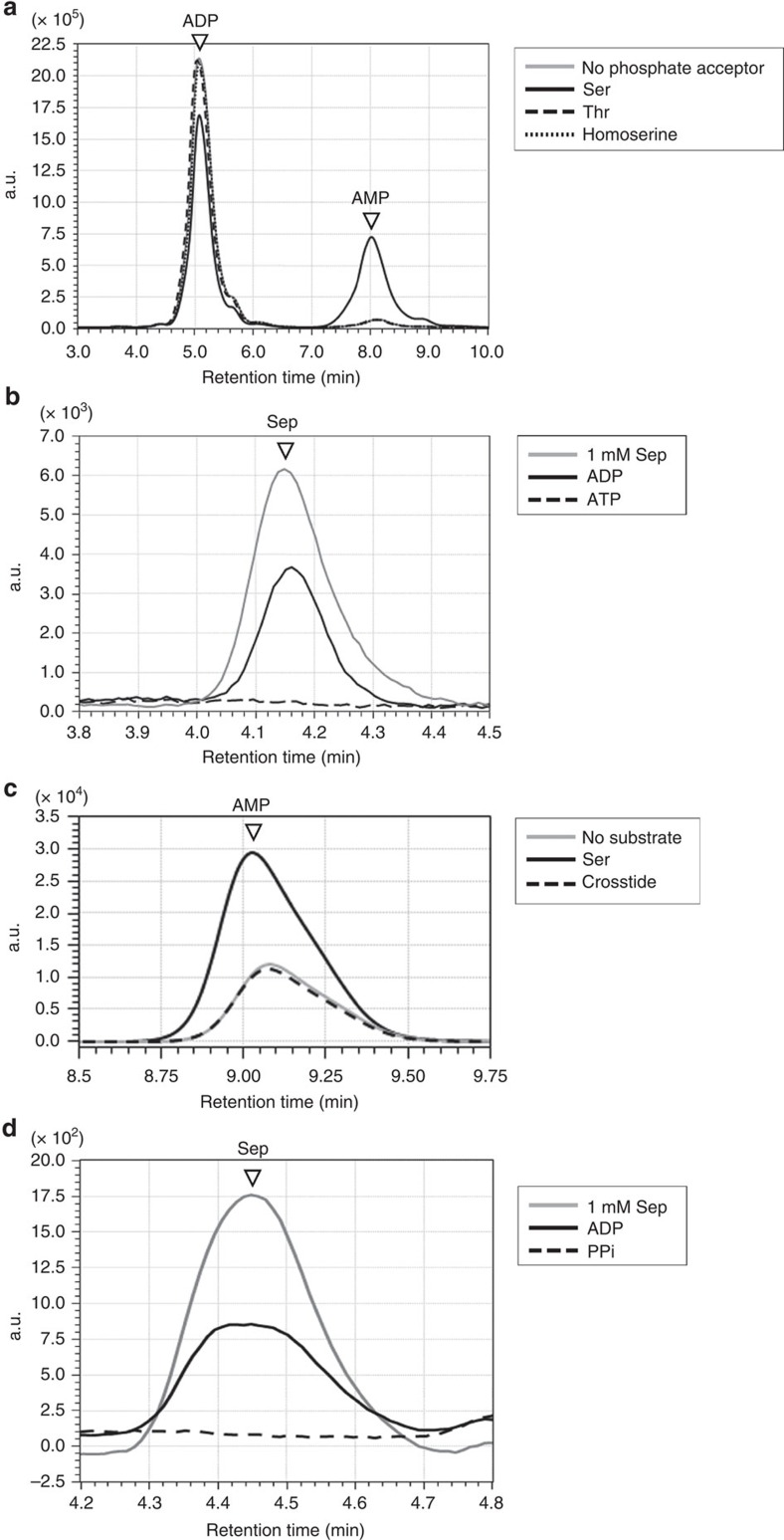
ADP-dependent Ser kinase activity and substrate specificity of the TK0378 protein. ADP-dependent Ser kinase activity of recombinant TK0378 protein was examined. Phosphate acceptor specificity (**a**,**c**) and phosphate donor specificity (**b**,**d**) were investigated. (**a**) Grey line, no phosphate acceptor; black solid line, 50 mM Ser; black broken line, 50 mM Thr; black dotted line, 50 mM homoserine. (**b**) Black solid line, 20 mM ADP; black broken line, 20 mM ATP; grey line, 1 mM standard Sep. (**c**) Grey line, no substrate; black solid line, 50 mM Ser; black broken line, 100 μM Crossitide (a substrate for Ser protein kinase). (**d**) As 20 mM pyrophosphate precipitated in reaction mixture, phosphate donors were applied here at 0.5 mM. Black solid line, 0.5 mM ADP; black broken line, 0.5 mM pyrophosphate; grey line, 1 mM standard *O*-phosphoserine (Sep).

The recombinant proteins of TK0378, TK1110 and TK1998 were produced in *E. coli* and partially purified by heat treatment (Supplementary Fig. 7). ADP-dependent Ser kinase activities of the three recombinant proteins were examined by HPLC. To our surprise, only TK0378, and not TK1110, exhibited significant ADP-dependent Ser kinase activity (Supplementary Fig. 8a–c). Therefore, TK0378 was further purified (Supplementary Fig. 9) and examined. In addition to AMP production from ADP (Fig. 3a) (286±36 μmol min^−1^ mg^−1^), we confirmed the generation of Sep from Ser in the TK0378 protein reaction (Fig. 3b). The substrate specificity of the TK0378 protein was then investigated. Thr and homoserine were not recognized as a phosphate acceptor (Fig. 3a). In addition, the TK0378 protein did not recognize Ser residues in Crosstide, an eleven-residue peptide which is known to be a substrate for human RAC-α serine/threonine-protein kinase (Akt1) (Fig. 3c). Akt1 recognized Crosstide protein as a substrate, but did not exhibit kinase activity towards free Ser (Supplementary Fig. 10). In terms of the phosphate donor, ATP and pyrophosphate were not utilized (Fig. 3b,d). The kinetic parameters of the TK0378 reaction with Ser and ADP were determined (Supplementary Fig. 11) (*k*_cat_: 218±5 s^−1^, *K*_*m*_: 5.1±0.5 mM, and *k*_cat_/*K*_*m*_: 42.4 mM^−1^s^−1^ towards Ser and *k*_cat_: 204±12 s^−1^, *K*_*m*_: 2.4±0.5 mM, and *k*_cat_/*K*_*m*_: 86.4 mM^−1^s^−1^ towards ADP). These results clearly revealed that TK0378, which had been annotated as a chromosome partitioning protein ParB, encoded an ADP-dependent Ser kinase (SerK).

### Physiological function of ADP-dependent Ser kinase

In order to examine the physiological function of the ADP-dependent Ser kinase, the TK0378 gene was disrupted and the growth properties of the resulting TK0378 mutant (Δ*serK*) were evaluated. Although the disruption of TK0378 alone did not lead to complete Cys auxotrophy, a clear growth defect was observed in a medium without Cys (Fig. 2g,h). This result indicates that *serK* actually contributes to Cys biosynthesis. The result also implies the presence of another pathway for Cys generation independent of *serK*. We thus combined the *serK* disruption with other gene disruptions blocking other possible pathways. Using the Δ*tk1449* strain and Δ*serA*Δ*ldhA1*Δ*ldhA2* strain as host strains, *serK* gene disruption was additionally carried out. In the former mutant (Δ*tk1449*Δ*serK*), the metabolic routes to Cys from Met via homocysteine and cystathionine (pathway Cys1) and from Ser via Sep (pathway identified in this study) were shut down. In the latter mutant (Δ*serA*Δ*ldhA1*Δ*ldhA2*Δ*serK*), the metabolic routes to Cys from 3-phosphoglycerate via Sep (pathway Cys2) and from Ser via Sep were interrupted. The Δ*tk1449*-Δ*serK* double mutant showed growth comparable to that of the Δ*serK* single mutant (Fig. 2g,h), implying that the metabolic route from Met to Cys is not the pathway supporting Cys biosynthesis in the Δ*serK* strain. On the other hand, the Δ*serA*-Δ*ldhA1*-Δ*ldhA2*-Δ*serK* multiple disruptant exhibited complete Cys auxotrophy (Fig. 2g,h), whereas the Δ*serA*-Δ*ldhA1*-Δ*ldhA2* mutant displayed growth in the absence of Cys (Fig. 2a,b).

**Figure 4:**
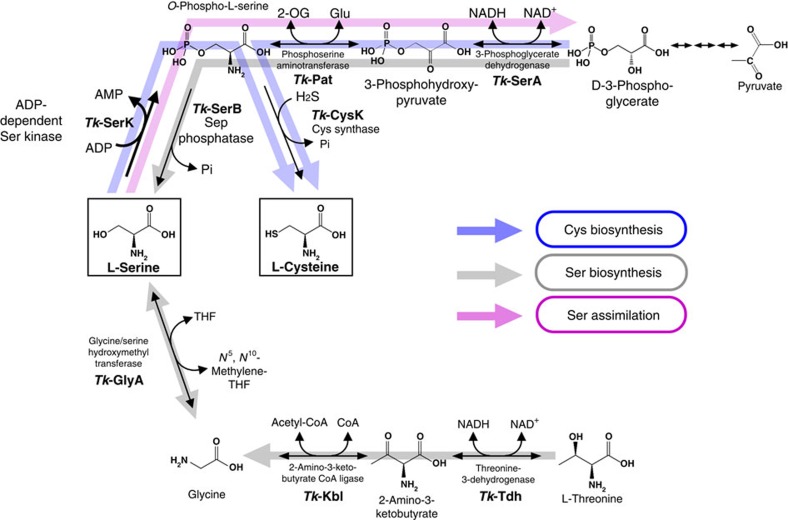
Predicted pathways for Cys and Ser metabolism in *Thermococcus kodakarensis*. Pathways involved in Cys biosynthesis, Ser biosynthesis and Ser assimilation identified in this study are highlighted with blue, grey and pink arrows, respectively. Cys can be generated from Ser or 3-phosphoglycerate. Ser can be generated from Thr (via Gly) or 3-phosphoglycerate. Ser can be assimilated to 3-phosphoglycerate. The SerK reaction is indicated with a thick arrow. THF, tetrahydrofolate.

The results obtained here indicate that there are two metabolic routes for Cys biosynthesis in *T. kodakarensis*, one from 3-phosphoglycerate and the other from Ser via SerK (Fig. 4). Among the three genes *serA*, *ldhA1* and *ldhA2*, we show below that *serA* is the gene involved in the route from 3-phosphoglycerate. Under the applied growth conditions, Cys biosynthesis from Ser is predominant, and there are no other routes that generate Cys, at least to the extent to support growth. The route from Ser is a previously unrecognized route for Cys biosynthesis utilizing a phosphorylation reaction instead of the acetylation reaction, observed in bacteria and plants, to activate the Ser side chain.

### Identification of a pathway involved in Ser biosynthesis

**Figure 5:**
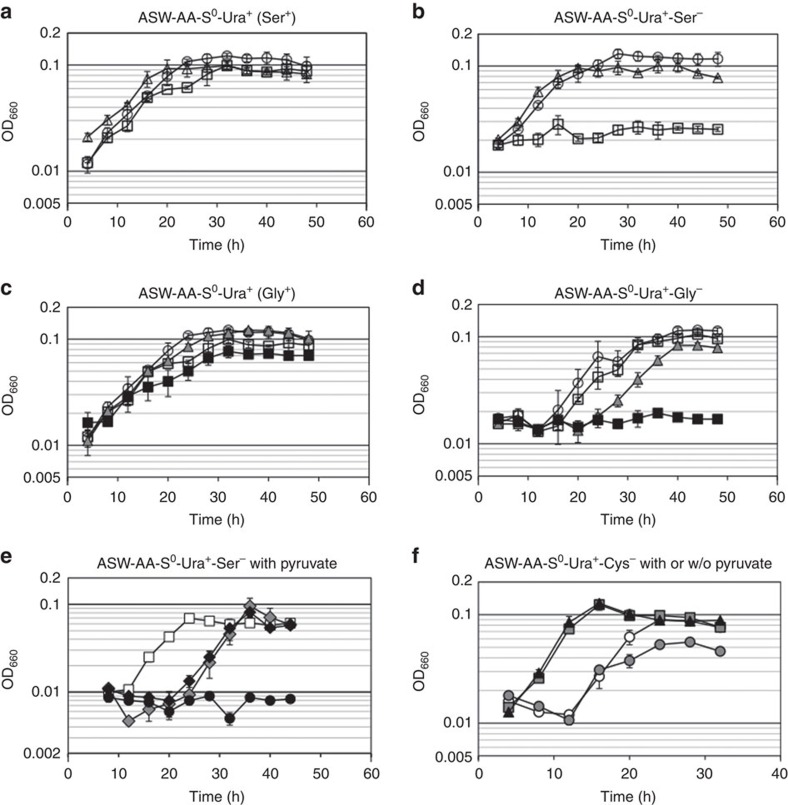
Ser, Gly or Cys auxotrophy of *Thermococcus kodakarensis* gene disruptants. Ser or Gly auxotrophy was investigated by cultivating cells in synthetic amino acid medium with (**a**) or without Ser (**b**) or with (**c**) or without Gly (**d**). Symbols: *T. kodakarensis* KU216 (open circles), Δ*serA*Δ*ldhA1*Δ*ldhA2*-1 (open triangles), Δ*glyA* (open squares), Δ*tdh* (grey triangles), Δ*glyA*Δ*tdh* (black squares). Contribution of the route from 3-phosphoglycerate to Ser biosynthesis was examined in synthetic amino acid medium supplemented with pyruvate and depleted of Ser (**e**). Contribution of the route from 3-phosphoglycerate to Cys biosynthesis was examined in synthetic amino acid medium depleted of Cys with or without pyruvate (**f**). (**e**) Symbols: Δ*glyA* (open squares), Δ*glyA*Δ*serA* (black circles), Δ*glyA*Δ*ldhA1* (grey diamonds), Δ*glyA*Δ*ldhA2* (black diamonds). (**f**) Symbols: KU216 host strain (open circles), Δ*serK* (grey circles), KU216 host strain with pyruvate (grey squares), Δ*serK* with pyruvate (black triangles). Error bars indicate the standard deviations of three independent culture experiments.

*T. kodakarensis* can grow in a synthetic amino acid medium without Ser (Fig. 5a,b), indicating that this organism can also synthesize Ser from the other 19 amino acids. As a pathway that generates Cys from Ser was identified, we further examined the pathways responsible for the biosynthesis of Ser. There are three established pathways that result in the biosynthesis of Ser (Fig. 1b). One pathway, reported in microorganisms, mammals and plants, generates Ser from 3-phosphoglycerate via Sep, and is called the ‘phosphorylated pathway' for Ser biosynthesis (pathway Ser1) (refs 33, 34, 35, 36, 37). The route from 3-phosphoglycerate to Sep is also utilized for Cys biosynthesis (for example, pathway Cys2 in Fig. 1a). Sep is converted to Ser by phosphoserine phosphatase (SerB). The second pathway is the ‘non-phosphorylated pathway' (pathway Ser2), which is utilized by eukaryotes. Ser is generated from 3-phosphoglycerate via glycerate and hydroxypyruvate, and involves 3-phosphoglycerate phosphatase, glycerate dehydrogenase and serine-pyruvate aminotransferase^38^. In the third pathway which is found in microorganisms and plants, Ser is generated directly from Gly through the function of glycine/serine hydroxymethyltransferase (GlyA) (pathway Ser3). This conversion is included in the serine pathway in methanotrophic and methyltrophic bacteria which utilize C1 compounds as a carbon source^39^.

A genetic approach was taken to determine the routes involved in Ser biosynthesis in *T. kodakarensis*. In the case of the triple gene disruption strain (Δ*serA* Δ*ldhA1* Δ*ldhA2*) in which pathway Ser1 was blocked, growth in medium depleted of Ser was similar to that of the host strain (Fig. 5a,b), suggesting that this route is not the main pathway for Ser biosynthesis from amino acids. In terms of pathway Ser2, genes annotated as 3-phosphoglycerate phosphatase and glycerate dehydrogenase were not present on the *T. kodakarensis* genome. In contrast, the *glyA* (TK0528) gene disruption strain (Δ*glyA*) could not grow at all without Ser (Fig. 5a,b), indicating that the GlyA function in pathway Ser3 is necessary for Ser biosynthesis, and that Ser is generated from Gly under these conditions. As the GlyA reaction has been reported to function for the generation of Gly from Ser in a number of organisms^40^,^41^,^42^, we examined whether this is also the case in *T. kodakarensis*. The *glyA* gene disruption strain was grown in a synthetic amino acid medium depleted of Gly but with Ser. As shown in Fig. 5c,d, growth of the disruption strain was similar to that of the host strain. Based on genome sequence data, we identified a possible route for Gly biosynthesis from Thr, consisting of threonine-3-dehydrogenase (Tdh, TK0916), which has been characterized *in vitro*^43^, and 2-amino-3-ketobutyrate CoA ligase (Kbl, TK2217) (Fig. 4). A Δ*tdh* mutant strain devoid of TK0916, catalysing the reaction from Thr to 2-amino-3-ketobutyrate, displayed growth defects in a medium without Gly (Fig. 5c,d). In addition, a Δ*tdh* and Δ*glyA* double mutant showed complete Gly auxotrophy (Fig. 5c,d). These results suggest that Gly is mainly synthesized from Thr in this medium, and that *glyA* also contributes to Gly synthesis (from Ser), but to a lower extent (Fig. 4). It should be noted that even *T. kodakarensis* KOD1, the wild-type strain, displays Thr auxotrophy, and that the presence of excess Gly does not complement this auxotrophy. In summary, the results indicate that in *T. kodakarensis*, Thr is an essential amino acid, a unidirectional route exists from Thr to generate Gly, and Ser and Gly can be converted to one another via the GlyA reaction (Fig. 4).

### Ser and Cys biosyntheses from 3-phosphoglycerate

We have experimentally demonstrated that a pathway is present from 3-phosphoglycerate to Sep in *T. kodakarensis*. As a SerB homolog, which catalyses the dephosphorylation of Sep to Ser, is also present on the genome, it was slightly unexpected that the single disruption of *glyA* resulted in Ser auxotrophy and that the triple gene disruption of *serA-ldhA1*-*ldhA2* did not affect the growth in medium without Ser. In order to examine whether this was simply due to the lack of starting material, we added pyruvate (final concentration: 0.5%) to the medium in order to replenish the supply of 3-phosphoglycerate. As a result, we found that the addition of pyruvate complemented the Ser auxotrophy of the *glyA* disruptant (Fig. 5e). These results suggest that Ser biosynthesis is possible from both Gly (via *glyA*) and 3-phosphoglycerate depending on their relative abundance (Fig. 4).

As described above, the *serK* disruptant displayed a growth defect in an amino acid medium depleted of Cys. In the presence of pyruvate, however, the growth retardation of the *serK* disruptant was not observed (Fig. 5f), suggesting that Cys biosynthesis from 3-phosphoglycerate (pathway Cys2 in Fig. 1a) is also relevant *in vivo* when the supply of 3-phosphoglycerate is sufficient. This indicates that Cys biosynthesis is possible from both Ser (via *serK*) and 3-phosphoglycerate (via *serA*, see next section) (Fig. 4).

### Identification of the 3-phosphoglycerate dehydrogenase

The complementation of Ser auxotrophy with pyruvate provided us with a growth condition to compare the contribution of the three gene candidates (*serA*, *ldhA1* and *ldhA2*) towards 3-phosphoglycerate dehydrogenase activity *in vivo*. In a *glyA* disruption background, the three genes were individually deleted, resulting in the construction of three mutants with the genotypes (Δ*glyA* Δ*serA*), (Δ*glyA* Δ*ldhA1*) and (Δ*glyA* Δ*ldhA2*). Their growth properties were examined in a synthetic amino acid medium depleted of Ser and supplemented with pyruvate. As a result, Δ*glyA*-Δ*ldhA1* and Δ*glyA*-Δ*ldhA2* mutants could grow, whereas the disruption of *serA* in addition to *glyA* abolished the complementation effect by pyruvate (Fig. 5e), revealing that *serA* (TK1966) is the 3-phosphoglycerate dehydrogenase in *T. kodakarensis*, and that the cysteine auxotrophy observed in the Δ*serA*Δ*ldhA1*Δ*ldhA2*Δ*serK*-1 strain (Fig. 2h) was due to the disruption of *serA* and *serK*.

### SerK-dependent Ser metabolism in *Thermococcus*

As described above, 3-phosphoglycerate dehydrogenase (TK1966) was shown to function in the pathway directing 3-phosphoglycerate to Ser and Cys via Sep. If there were a sufficient supply of an amino acceptor for the aminotransferase (TK1548) reaction and NADH for the 3-phosphoglycerate dehydrogenase reaction, the Ser kinase reaction may allow the conversion of Ser to 3-phosphoglycerate, in addition to the conversion of Ser to Cys. Indeed, as shown in Fig. 2g, we observed a small decrease in cell yield in the Δ*serK* strain compared with the host strain in synthetic medium containing all amino acids. We confirmed this small, but consistent decrease in cell yield in three additional growth experiments (Supplementary Fig. 12a) as well as in the presence of higher concentrations of Ser (Supplementary Fig. 12b,c). This decrease was also observed in the Δ*serK*Δ*tk1449* double mutant and Δ*serK*Δ*serA*Δ*ldhA1*Δ*ldhA2* strain (Fig. 2g), whereas it was not observed in the Δ*tk1449* strain (Fig. 2a,c) and the Δ*cysK* strain (Fig. 2e). The fact that no change was detected in the growth of the Δ*cysK* strain indicated that the decrease in cell yield in the Δ*serK* strain was not due to the lack of supply of Cys. If the decrease in cell yield in the Δ*serK* strain were due to the shutdown in the pathway from Ser to 3-phosphoglycerate via Sep, we should also see the same decrease in cell yield in strains deleted of *serA*. This was the case, as the Δ*serA*Δ*ldhA1*Δ*ldhA2* strain and the Δ*tk1449*Δ*serA*Δ*ldhA1*Δ*ldhA2* strain both displayed lower cell yield than the host strain and the Δ*tk1449* strain (Fig. 2a). The capacity of this pathway does not seem to be able to accommodate higher concentrations of Ser, as increasing the Ser concentration in the medium 2- or 3- fold does not increase the cell yield of the host strain (Supplementary Fig. 12d). Increasing the concentrations of Ser further led to growth inhibition. The supply of Ser for cell growth can be considered to be already saturated in the host strain without exogenous Ser in the medium. There is no difference in growth of the host strain in the presence or absence of Ser in the medium (Supplementary Fig. 12e), as long as the *glyA* gene is intact and Gly is present in the medium. These results suggest that Ser is sufficiently supplied from Gly. Supporting this, the Δ*glyA* and Δ*glyA*Δ*tdh* strains also displayed a small decrease in cell yield compared with that of the host strain (Fig. 5c). As the effects on growth brought about by disruption of *serA*, *serK* or *glyA* were relatively small, we cannot rule out other possibilities, but the results raise the possibility that the phosphorylation of free serine provides a previously unrecognized route for Ser assimilation (Fig. 4) in addition to Cys biosynthesis. The route, along with GlyA, can also be utilized for Gly assimilation.

## Discussion

Here we have identified SerK, an archaeal ADP-dependent kinase that phosphorylates free Ser. The SerK reaction initiates a previously unrecognized route from Ser to Cys. Our results also suggest that the reaction is the first step of a pathway metabolizing Ser to 3-phosphoglycerate, an intermediate of central sugar metabolism. Closely related homologs of *serK* are distributed throughout the *Thermococcus* and *Pyrococcus*, and are also found in the *Desulfurococcus* and *Staphylothermus*. Clear-cut homologs of SerK are not found on other archaeal genomes and those from bacteria and eukaryotes. The four genera are known as typical representatives of heterotrophic, hyperthermophilic, sulfur-reducing archaea, with the former two genera belonging to the Euryarchaeota and the latter two the Crenarchaeota. The Thermococcales are well known for their ability to utilize amino acids and peptides as a source of carbon and energy. The ability to produce Cys from Ser, in addition to the well-known pathway from 3-phosphoglycerate, may thus provide an advantage to these organisms when utilizing amino acids. The capability to direct the carbon from Ser to glycolysis and gluconeogenesis should also provide an advantage to utilize Ser (and Gly and Thr). Concerning the two genera of Crenarchaeota, *Desulfurococcus mucosus* has been reported to utilize casein and its tryptic digest, but not sugars^44^, a trait in which SerK-dependent metabolism would provide an advantage. *Staphylothermus marinus* has also been shown to display growth on peptides as carbon and energy source^45^. The presence of SerK in these peptide-utilizing organisms can be expected to facilitate the efficient utilization of amino acids. Another reason why SerK is present only in some hyperthermophiles and not in mesophiles may be that Sep is more thermostable than *O*-acetylserine, which has been discussed elsewhere^16^. Cys biosynthesis from Ser through Sep may represent a means to avoid thermal degradation of *O*-acetylserine, which may not be necessary in mesophiles.

In addition to the phosphorylation of free Ser, the utilization of ADP as a phosphate donor is a notable feature of *Tk*-SerK. It is known that some archaeal phosphofructokinases and glucokinases and eukaryotic phosphofructokinases utilize ADP as the phosphate donor instead of ATP^3^,^46^. Recently, an ADP-dependent ribose-1-phosphate kinase was also identified in *T. kodakarensis*^14^. ADP-dependent kinases are considered atypical, and their occurrence has been limited to sugar kinases. The identification of the ADP-dependent SerK, which recognizes a non-sugar substrate, Ser, raises the possibilities that ADP-dependent kinases may be more widely distributed than have been recognized until now.

Although we have tried to identify kinases with similarity to *Tk*-SerK, no sequences confirmed to be or annotated as kinases were found at least up to a 0.001-value in a BLAST search (Supplementary Table 3). Even when we compare the primary structure of *Tk*-SerK with other ADP-dependent kinases, *Tk*-SerK is only 21.5% identical to ADP-dependent glucokinase (TK1110), 20.7% identical to ADP-dependent phosphofructokinase (TK0376) and 18.6% identical to ADP-dependent ribose-1-phosphate kinase (TK2029) from *T. kodakarensis*. These results suggest that TK0378 homologs form a group of kinases distinct to previously recognized kinase families, including ADP-dependent sugar kinases. The protein also displays only limited similarity towards experimentally examined bacterial ParB proteins^30^; from *Bacillus subtilis* (BSU40960: 18.6% identical), *Caulobacter crescentus* (CC_3752: 17.4% identical), *Streptomyces coelicolor* (SCO1789: 20.7% identical) and *Corynebacterium glutamicum* (NCgl2988: 18.6% identical).

As our genetic analyses suggested that SerK was involved in the assimilation of Ser, including Cys biosynthesis, we examined the location of *serK* on the genomes of various members of the Thermococcales and Desulfurococcales. The *serK* gene in *T. kodakarensis* does not constitute an operon with other genes. In other members of the Thermococcales, we do not observe a tendency of the *serK* genes to cluster with the aminotransferase and 3-phosphoglycerate dehydrogenase genes. On the other hand, on the genome of three Desulfurococcales species, *D. mucosus*, *S. marinus* and *Staphylothermus hellenicus*, *serK* genes are located nearby genes annotated as 3-phosphoglycerate dehydrogenase (or D-isomer specific 2-hydroxyacid dehydrogenase) and aminotransferase (Supplementary Fig. 13a). In *Desulfurococcus kamchatkensis*, the *serK* homolog is adjacent to the 3-phosphoglycerate dehydrogenase homolog. Some of these genes in the Desulfurococcales species seem to form an operon, supporting the presence of Ser assimilation pathways involving Ser kinase. Based on the presence of *serK* homologs and the presence/absence of the insertion sequence in CysK, we can assume that *D. mucosus* and *S. marinus* (with the insertion sequence in CysK, Supplementary Fig. 13a) can also metabolize Ser to both Cys and 3-phosphoglycerate, while *D. kamchatkensis* and *S. hellenicus*, (apparently without the insertion sequence in CysK) convert Ser to only 3-phosphoglycerate (Supplementary Fig. 13b).

In respect to Ser metabolism, routes that direct Ser carbon to central sugar metabolism are limited. The most direct link is deamination catalysed by Ser dehydratase converting Ser to pyruvate and ammonium. In addition, it is considered that the non-phosphorylated pathway can function as a Ser assimilation pathway^35^ by converting Ser to hydroxypyruvate and then to glycerate followed by the phosphorylation of glycerate to phosphoglycerate by glycerate kinase. Although the 3-phosphoglycerate dehydrogenase and phosphoserine aminotransferase in pathway Ser1 (Fig. 1b) have been recognized as enzymes contributing to Ser biosynthesis, the identification of Ser kinase raises the possibility that these enzymes, along with SerK, function in Ser assimilation (Fig. 4). The decreases in cell yield observed in all strains with deletions in *serK*, *serA*, and/or *glyA* support this notion, at least in *T. kodakarensis*.

Anaerobic heterotrophic archaea have attracted attention in relation with the origin of heterotrophic life, and several pathways have been focused upon as ancient mechanisms to metabolize amino acids and pentoses^47^. The SerK-dependent pathway, present in the Thermococcales and most likely also in the Desulfurococcus, both of which are anaerobic, heterotrophic, peptide-assimilating archaea, may represent another example of an ancient heterotrophic mechanism to make use of Ser.

## Methods

### Strains and medium

The strains and plasmids used in this study are listed in Supplementary Table 1. *T. kodakarensis* was cultivated under strictly anaerobic conditions at 85 °C in a nutrient-rich medium (ASW-YT-S^0^) and a synthetic medium (ASW-AA-S^0^). ASW-YT-S^0^ medium was composed of 0.8 × ASW^24^, 5.0 gl^−1^ yeast extract, 5.0 gl^−1^ tryptone and 2 gl^−1^ elemental sulfur. ASW-AA-S^0^ medium was composed of 0.8 × ASW, a mixture of 20 amino acids, minerals, a mixture of vitamins and 2 gl^−1^ elemental sulfur^24^. When appropriate, sodium pyruvate was added at a concentration of 5.0 gl^−1^. Solid medium used to isolate transformants were based on ASW-AA-S^0^ medium supplemented with 10 gl^−1^ gelrite, 7.5 gl^−1^ 5-fluoroorotic acid (5-FOA), 10 μg ml^−1^ of uracil, 4.5 ml of 1 M NaOH and 0.2% (v/v) polysulfide solution^24^ instead of elemental sulfur. *Escherichia coli* DH5α and BL21-CodonPlus(DE3)-RIL strains were used for plasmid construction and heterologous gene expression, respectively. These strains were cultivated at 37 °C in Luria-Bertani medium containing 50 μg ml^−1^ ampicillin.

### Construction of gene disruption vectors

To construct disruption vectors for *tk1449* (TK1449, *metC*), *Tk*-*serA* (TK1966), *Tk*-*ldhA1* (TK0551), *Tk*-*ldhA2* (TK0683), *Tk-cysK* (TK1687), *Tk-serK* (TK0378), *Tk-glyA* (TK0528) and *Tk-tdh* (TK0916), their coding regions along with about 1,000 bps of 5′- and 3′-flanking regions were amplified by PCR using *T. kodakarensis* KOD1 genomic DNA as a template. In the case of all vectors other than the *Tk-cysK* disruption vector, the amplified fragments were inserted into the HincII restriction site of the plasmid pUD3, harbouring a *pyrF* marker cassette^7^. Inverse PCR was performed in order to remove the respective coding regions of target genes and the amplified fragments were self-ligated. For the *Tk-cysK* disruption vector, the amplified coding region along with 5′- and 3′-flanking regions was ligated with pUC118 digested with HincII. After inverse PCR to exclude the target gene, the fragment was ligated with the *pyrF* marker gene cassette excised from pUD2 (ref. 25) by PvuII. The resulting eight disruption vectors were sequenced and confirmed to have no unintended mutations. Primers used for disruption vector construction are listed in Supplementary Table 4.

### Transformation of *T. kodakarensis*

To construct gene disruption strains, *T. kodakarensis* KU216 (Δ*pyrF*) was used as the host strain. Transformation of *T. kodakarensis* was performed as follows^24^,^25^. Cells grown in ASW-YT-S^0^ rich medium for 12 h were harvested (4 °C, 5,400*g*, 10 min), resuspended in 200 μl of 0.8 × ASW, and kept on ice for 30 min. The disruption vector (3 μg) was added to the cells and the mixture was kept on ice for 1 h. After heat-shock at 85 °C for 45 s, the mixtures were kept on ice for 10 min. The cells were subcultured in ASW-AA-S^0^ synthetic medium and incubated at 85 °C for 2 days. Cells were subcultured in the same medium and cultivated for another 2 days in order to enrich the transformants which harbour the *pyrF* gene due to single crossover insertion (pop-in recombination). The cells were spread onto ASW-AA solid medium with 0.75% 5-FOA and 10 μg ml^−1^ uracil. After incubation at 85  °C for 3–5 days, cells in which the *pyrF* gene was removed via a second recombination event (pop-out recombination) were selected under the presence of 5-FOA. The genotypes of transformants were confirmed by PCR and DNA sequencing analyses. Primers used for analyses of deletion mutants are listed in Supplementary Table 4. In order to construct strains with multiple gene disruptions, the transformants and the *pyrF* gene were used as the host and selectable marker, respectively. In the case of *cysK* gene disruption, *pyrF* marker gene cassette was simply inserted into the *cysK* locus without pop-out recombination. Regarding all gene disruptions except for Δ*tdh*, Δ*glyA*Δ*serA*, Δ*glyA*Δ*ldhA1* and Δ*glyA*Δ*ldhA2* mutants, two mutant strains were obtained. Among them, one mutant was used for growth experiments.

### Investigations of growth properties of gene disruptants

Amino acid auxotrophy of gene disruption strains was examined using a synthetic ASW-AA-S^0^ medium supplemented with 5 μg ml^−1^ uracil and 10 μM of tungsten (ASW-AA-S^0^-Ura^+^). Gene disruptants were pre-cultivated in ASW-AA-S^0^-Ura^+^ medium and its cell densities were determined by measuring optical densities at 660 nm (OD_660_). The same number of cells were inoculated into ASW-AA-S^0^-Ura^+^ medium with or without Cys, Met, Ser or Gly and incubated at 85 °C. Growth curves were constructed based on the monitored OD_660_ values.

### Preparation of CFE

*T. kodakarensis* cells were cultivated in ASW-YT-Pyruvate medium for 18 h. Cells were harvested (4 °C, 6,000*g,* 20 min) and re-suspended in (1 per 60 volume of medium) 0.8 × ASW. After centrifugation (4 °C, 6,000*g,* 20 min) and removal of supernatant, cells were re-suspended in (1 per 500 volume of medium) 50 mM Tris-HCl (pH 7.5) with 1% Triton X-100. After vortexing for 30 min, the cell lysate was centrifuged (4 °C, 20,400*g,* 20 min). The resulting supernatant was used as the CFE.

### Activity measurement of Ser kinase

ATP-dependent Ser kinase activity in *T. kodakarensis* CFE was measured by quantifying Ser-dependent ADP generation with a pyruvate kinase/LDH coupling assay. The first reaction mixture (100 μl) was composed of CFE (100 μg), 50 mM L-serine (Nacalai Tesque, Kyoto, Japan), 1 mM ATP (Oriental Yeast, Tokyo, Japan), 2 mM MgCl_2_ and 50 mM Tris-HCl (pH 7.5). After pre-incubation at 85 °C for 3 min in the absence of ATP, the reaction was initiated by adding ATP. The reaction was carried out at 85 °C for 30 min and terminated by rapid cooling on ice for 10 min. Molecules with molecular weights more than 10,000 (for example, proteins, DNA and RNA) were removed by ultrafiltration with Amicon Ultra centrifugal filter unit (MWCO 10,000) (Millipore, Billerica, MA). An aliquot was applied to the second pyruvate kinase/LDH reaction mixture, which contained 5 mM phosphoenolpyruvate, 50 mM MES (pH 6.5), 0.2 mM NADH (Oriental Yeast), 7.4 U ml^−1^/9.3 U ml^−1^ pyruvate kinase/LDH enzymes from rabbit muscle (Sigma-Aldrich). The second reaction was started by the addition of the aliquot of first reaction mixture and the decrease in absorption at 340 nm was measured. The decrease in A_340_ in a control experiment without the addition of Ser in the first reaction mixture was subtracted from the results of each experiment.

ADP-dependent Ser kinase activity was measured by quantifying Ser-dependent AMP synthesis by HPLC (Shimadzu, Kyoto, Japan). When measuring activity in CFE, the reaction mixture (100 μl) was composed of 100 μg of CFE, 50 mM L-serine, 1 mM ADP, 2 mM MgCl_2_ and 50 mM Tris-HCl (pH 7.5). After pre-incubation at 85 °C for 3 min in the absence of ADP, the reaction was initiated by adding ADP. The reaction was carried out at 85 °C for 30 min and terminated by rapid cooling on ice for 10 min. After CFE was removed with Amicon Ultra centrifugal filter unit (MWCO 10,000), the filtrate was analysed by HPLC using COSMOSIL 5C18-PAQ column (Nacalai Tesque) with 50 mM sodium phosphate buffer (pH 4.4) as a mobile phase. The reaction products were detected by ultraviolet absorbance at 260 nm with SPD-20A detector (Shimadzu). When examining partially purified recombinant proteins of TK0378, TK1110 and TK1998, ADP-dependent Ser kinase activities were measured with a reaction time of 10 min. When examining purified recombinant TK0378 protein, 0.1 μg of purified protein was used and the concentrations of ADP and MgCl_2_ were increased to 20 mM each. The reaction was performed for 1, 2, and 3 min (to determine specific activity) or 10 min (to analyse reaction products). Phosphate donor specificity was investigated with 20 mM ATP or 0.5 mM pyrophosphate (PPi), while phosphate acceptor specificity was examined with 50 mM L-threonine or 50 mM L-homoserine. In addition, 100 μM crosstide (Merck, Darmstadt, Germany), eleven-residue peptide (Gly-Arg-Pro-Arg-Thr-Ser-Ser-Phe-Ala-Glu-Gly), was also tested as a substrate. When *O*-phosphoserine is detected and quantified for examination of phosphate donor specificity, 300 mM sodium phosphate buffer (pH 4.5) and NH2P-50 4E column (Shodex, Tokyo, Japan) were utilized as a mobile phase and a column, respectively. Ultraviolet absorbance at 210 nm was detected with SPD-20A detector.

### Activity measurement of Ser protein kinase AKT1

When detecting the activity of RAC-alpha Ser/Thr-protein kinase AKT1 derived from human, the reaction mixture (100 μl) was composed of 0.1 μg of AKT1, 100 μM L-serine or 100 μM Crosstide, 100 μM ATP, 10 mM MgCl_2_, and 50 mM Tris-HCl (pH 7.5). The reaction was performed at 24 °C for 30 min and finished by cooling on ice for 10 min. The produced ADP was detected by HPLC with the same method as in quantifying AMP mentioned above.

### Purification of serine kinase from CFE

All purification steps were performed at room temperature and all chromatography was performed with columns purchased from GE healthcare (Little Chalfont, Buckinghamshire, UK). CFE of *T. kodakarensis* prepared as described above was loaded onto an anion exchange column (Resource Q), which was equilibrated with 50 mM Tris-HCl (pH 7.5). Proteins were eluted with a linear gradient of 0 to 1.0 M NaCl. Fractions with high ADP-dependent Ser kinase activity were collected and adjusted to contain 1.5 M (NH_4_)_2_SO_4_. After centrifugation (4 °C, 20,400*g*, 30 min), the supernatant was applied to a hydrophobic column (Resource 15PHE) equilibrated with 1.5 M (NH_4_)_2_SO_4_ (pH 7.5). The bound proteins were eluted with a linear gradient of 1.5 to 0 M (NH_4_)_2_SO_4_ at pH 7.5. Fractions with high ADP-dependent Ser kinase activity were applied to a gel-filtration column (Superdex 200) equilibrated with 50 mM Tris-HCl (pH 7.5) containing 0.15 M NaCl. Separated fractions were examined for ADP-dependent Ser kinase activity.

### Liquid chromatography–mass spectrometry analysis

The fractions displaying ADP-dependent Ser kinase activity were applied to SDS– polyacrylamide gel electrophoresis (12.5% polyacrylamide) and silver stained. Proteins whose band intensities correlated with the levels of kinase activity were extracted from the gel with In-Gel Tryptic Digestion Kit (Thermo Fisher Scientific, San Jose, CA). HPLC was carried out with an Accela 600 series HPLC system equipped with an autosampler. The HPLC system was interfaced with an EXACTIVE (Thermo Fisher Scientific) Fourier-transfer mass spectrometer with an electrospray ionization source. Data acquisition and analysis were performed with Xcalibur software (version 2.2). Atlantis Silica HILIC Column (100 Å, 3 μm, 3 mm × 100 mm) (Waters, Milford, MA) was utilized. As mobile phase for HPLC, solution A: 100 mM ammonium acetate (pH 4.5) and acetonitrile (30:70 v/v), and solution B: acetonitrile were applied. The column was developed at a flow rate of 200 μl min^−1^ with the concentration gradient of acetonitrile: from 50% B to 0% B in 10 min, sustaining 0% B for 5 min, from 0% B to 50% B in 1 min, and finally re-equilibrating with 50% B for 10 min. The electrospray ionization source was operated in negative ion mode.

### Construction of expression vectors

TK0378, TK1110 and TK1998 coding regions with restriction sites were amplified by PCR using *T. kodakarensis* KOD1 genomic DNA as a template. After digestion by restriction enzymes, NdeI and EcoRI, the DNA fragments were ligated with pET21a(+) expression vector digested with the same enzymes. The resulting plasmids were designated as pET-TK0378, pET-TK1110 and pET-TK1998, respectively. The absence of unintended mutations was confirmed by DNA sequencing. Primers used for expression vector construction are listed in Supplementary Table 5.

### Preparation of partially purified recombinant proteins

*E. coli* strain BL21-CodonPlus(DE3)-RIL was transformed with pET-TK0378, pET-TK1110 and pET-TK1998. The transformants were cultivated at 37 °C in Luria-Bertani medium with ampicillin. After the OD_660_ reached 0.4–0.8, isopropyl-1-thio-β-D-galactopyranoside was added at a final concentration of 0.1 mM, and the culture was continued for another 4 h. Cells were harvested by centrifugation (4 °C, 5,000*g*, 20 min), washed with 50 mM Tris-HCl (pH 8.0) containing 1% NaCl, and collected by centrifugation (4 °C, 5,000*g*, 20 min). Cells were re-suspended with 50 mM Tris-HCl (pH 7.5) and sonicated (OUTPUT: 4, DUTY: 50, 10 min). After centrifugation (4 °C, 20,400*g*, 20 min), the supernatant was heat-treated for 10 min at 80 °C and centrifuged (4 °C, 20,400*g*, 30 min) to remove thermolabile proteins derived from the host cells.

### Purification of recombinant TK0378 protein

*E. coli* cells producing the recombinant TK0378 protein were harvested and sonicated as described above. After centrifugation (4 °C, 17,000*g*, 15 min), the supernatant was heat-treated at 85 °C for 10 min and centrifuged (4 °C, 17,000*g*, 15 min) to remove thermolabile proteins derived from the host cells. The supernatant was loaded onto an anion exchange column (Resource Q) equilibrated with 50 mM Tris-HCl (pH 7.5). Proteins were eluted with a linear gradient of 0 to 1.0 M NaCl. Fractions including the TK0378 protein were collected and concentrated with an Amicon Ultra centrifugal filter unit (MWCO 3,000). The resulting protein solution was applied to a gel-filtration column (Superdex 200) and proteins were separated with a mobile phase, 50 mM Tris-HCl (pH 7.5) containing 0.15 M NaCl.

### Data availability

The data that support the findings of this study are available from the corresponding author upon reasonable request.

## Additional information

**How to cite this article**: Makino, Y. *et al*. An archaeal ADP-dependent serine kinase involved in cysteine biosynthesis and serine metabolism. *Nat. Commun.* **7**, 13446 doi: 10.1038/ncomms13446 (2016).

**Publisher's note:** Springer Nature remains neutral with regard to jurisdictional claims in published maps and institutional affiliations.

## Supplementary Material

Supplementary Information Supplementary Figures 1-13, Supplementary Tables 1-5 and Supplementary References.
