# Supplementary material for: An archaeal ADP-dependent serine kinase involved in cysteine biosynthesis and serine metabolism
Source: Nat Commun. 2016 Nov 18;7:13446. doi: 10.1038/ncomms13446 (PMC5120207; doi:10.1038/ncomms13446)
Supplement: Supplementary Information — Supplementary Figures 1-13, Supplementary Tables 1-5 and Supplementary References. [file ncomms13446-s1.pdf]

Supplementary Figure 1

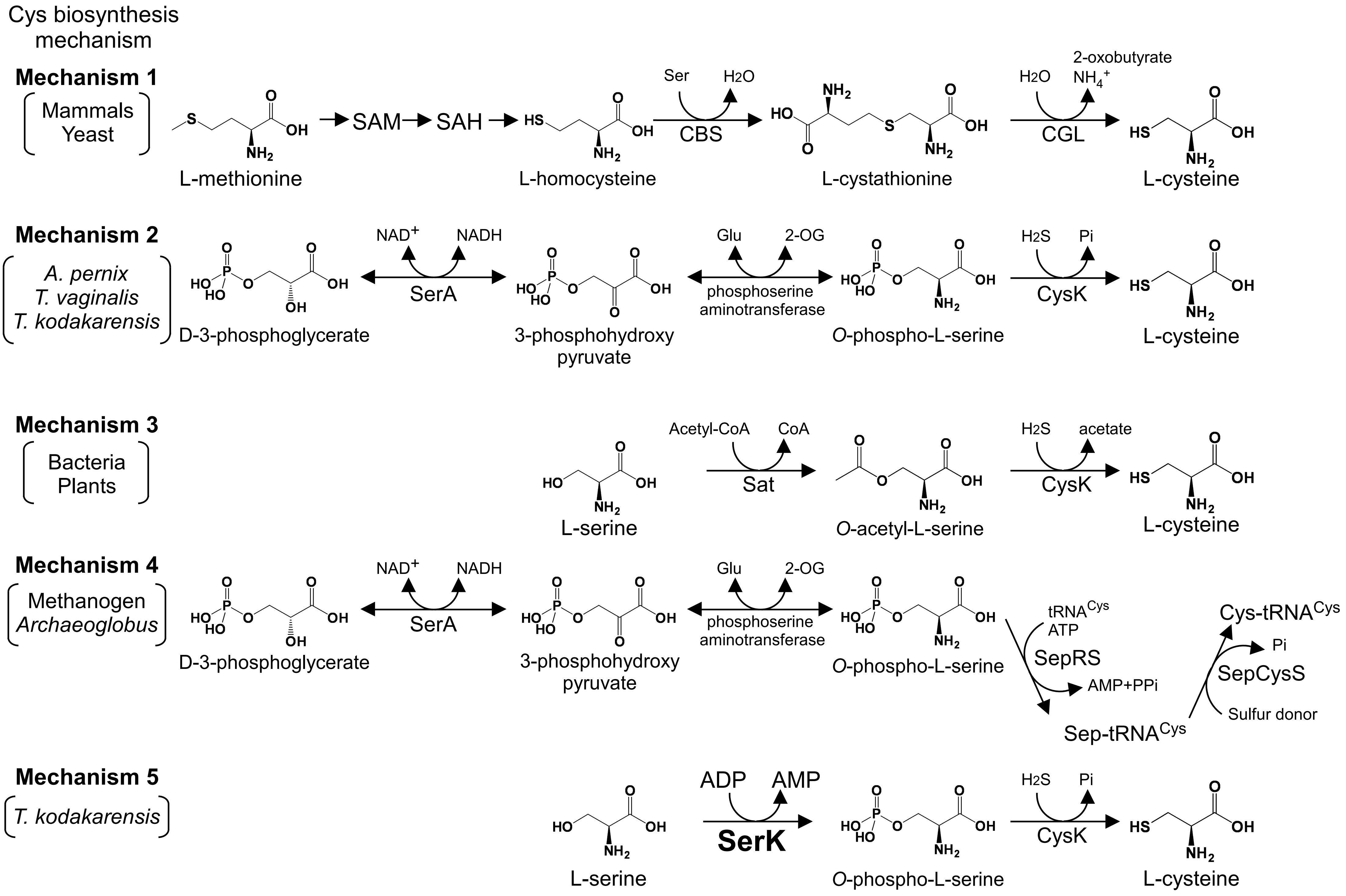

Supplementary Figure 1. The four previously recognized mechanisms for Cys biosynthesis and the Cys biosynthesis pathway identified in *Thermococcus kodakarensis*. Four mechanisms have been identified for Cys biosynthesis. Mechanism 1, found mainly in mammals and yeast[1], initiates from Met and leads to Cys via homocysteine. Mechanism 2 generates Cys from 3-phosphoglycerate via Sep, and is present in the hyperthermophilic archaeon *A. pernix*[2] and in protozoa *T. vaginalis*[3]. This study indicated that *T. kodakarensis* also utilizes this mechanism. Mechanism 3 is predominantly found in bacteria and plants and generates Cys from Ser via *O*-acetylserine[4,5]. Mechanism 4 is found in methanogenic archaea and *Archaeoglobus* in which Cys for protein synthesis is generated as cysteinyl tRNA from Sep-$tRNA^{Cys}$[6, 7]. The fifth mechanism for Cys biosynthesis in *T. kodakarensis* was identified in this study. Abbreviations; SAM: *S*-adenosylmethionine, SAH: *S*-adenosylhomocysteine, 2-OG: 2-oxoglutarate, Sep-$tRNA^{Cys}$: *O*-phosphoseryl-$tRNA^{Cys}$, Cys-$tRNA^{Cys}$: cysteinyl-$tRNA^{Cys}$, PPi: pyrophosphate and Pi: phosphate. Enzyme abbreviations; CBS: cystathionine β-synthase, CGL: cystathionine γ-lyase, CysK: cysteine synthase, Sat: serine acetyltransferase, SepRS: *O*-phosphoseryl-tRNA synthetase, SepCysS: Sep-tRNA:Cys-tRNA synthase, SerA: 3-phosphoglycerate dehydrogenase, SerK: serine kinase.

Supplementary Figure 2

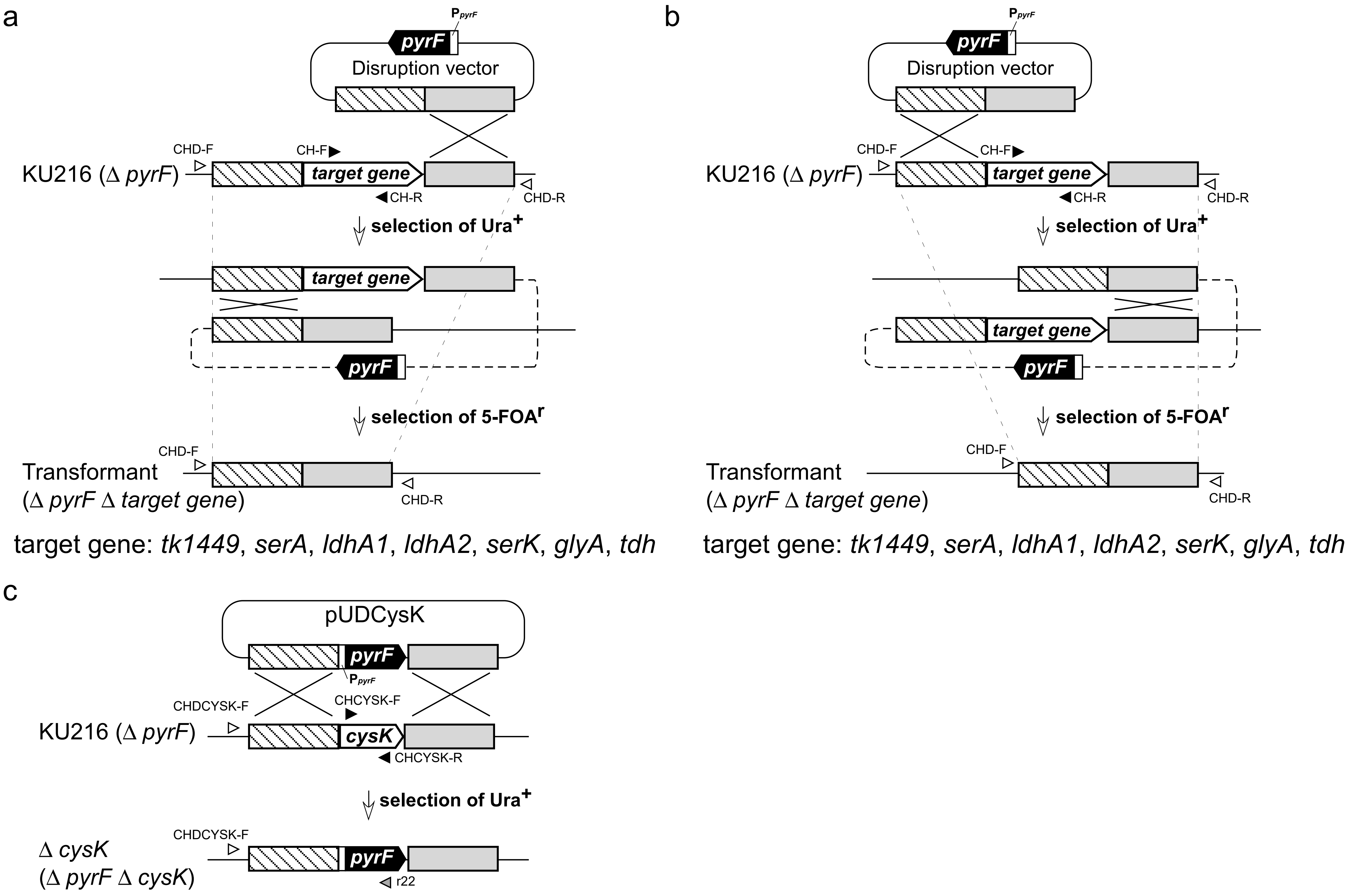

Supplementary Figure 2. Schematic drawing of gene disruptions in *T. kodakarensis*.

Gene disruptions of *tk1449*, *serA*, *ldhA1*, *ldhA2, serK, glyA* and *tdh* were carried out utilizing the pop-in/pop-out recombination strategies described in the Methods section **(a** or **b** occur**)**[8], while *cysK* gene disruption was performed by simply replacing the gene by a *pyrF* marker cassette **(c)**[8,9]. Diagonally striped and gray boxes denote 5'- and 3'-flanking regions of the target genes, which are the homologous regions for homologous recombination.

Open, closed and gray arrowheads indicate primers annealing outside of the 5'- and 3'-flanking regions of the target genes, within the target genes, and within the *pyrF* gene, respectively, which were used in the PCR analysis to confirm the genotypes of gene disruptants. P_{pyrF} represents the 5'-flanking region (putative promoter region) of the operon including the *pyrF* gene.

Supplementary Figure 3

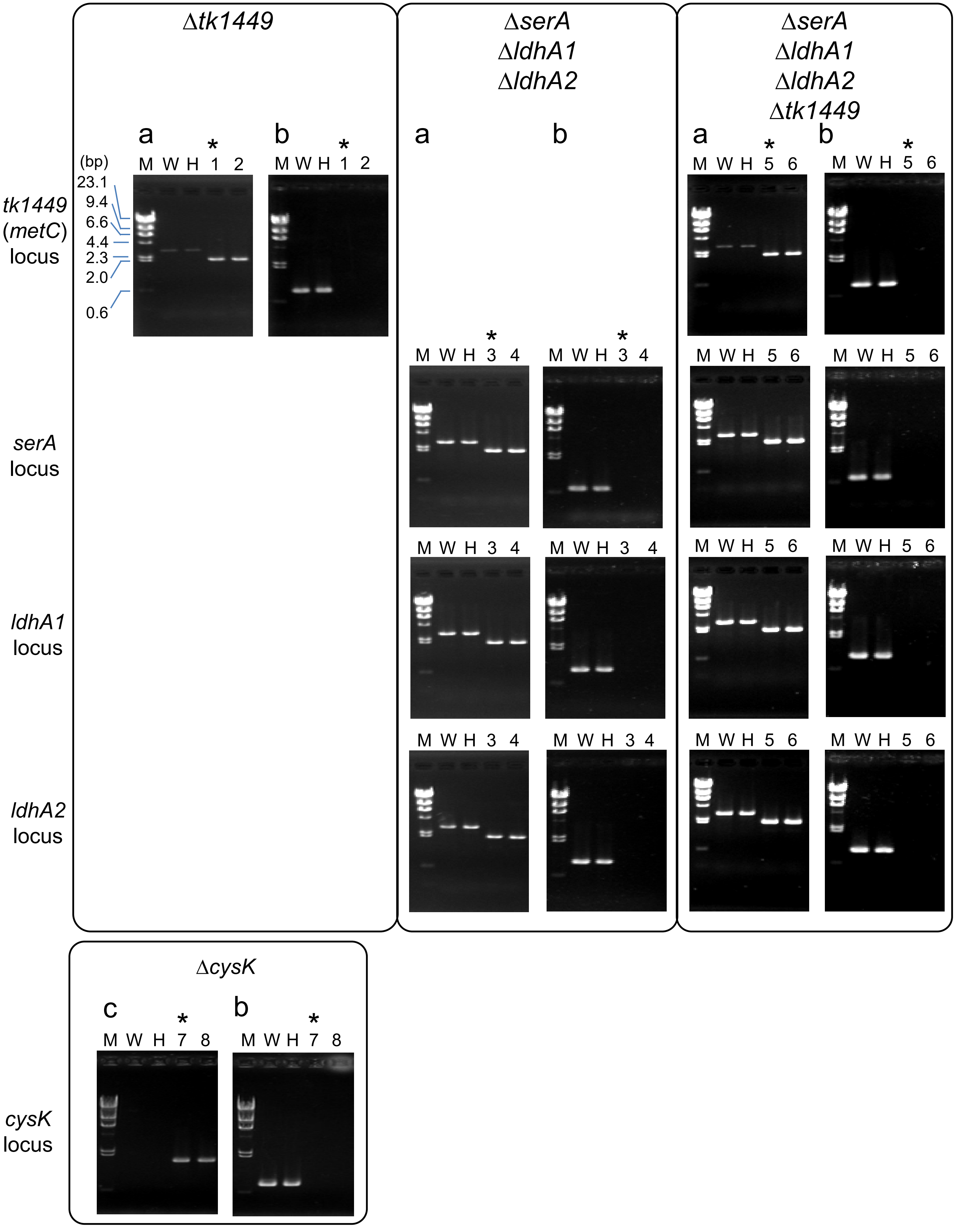

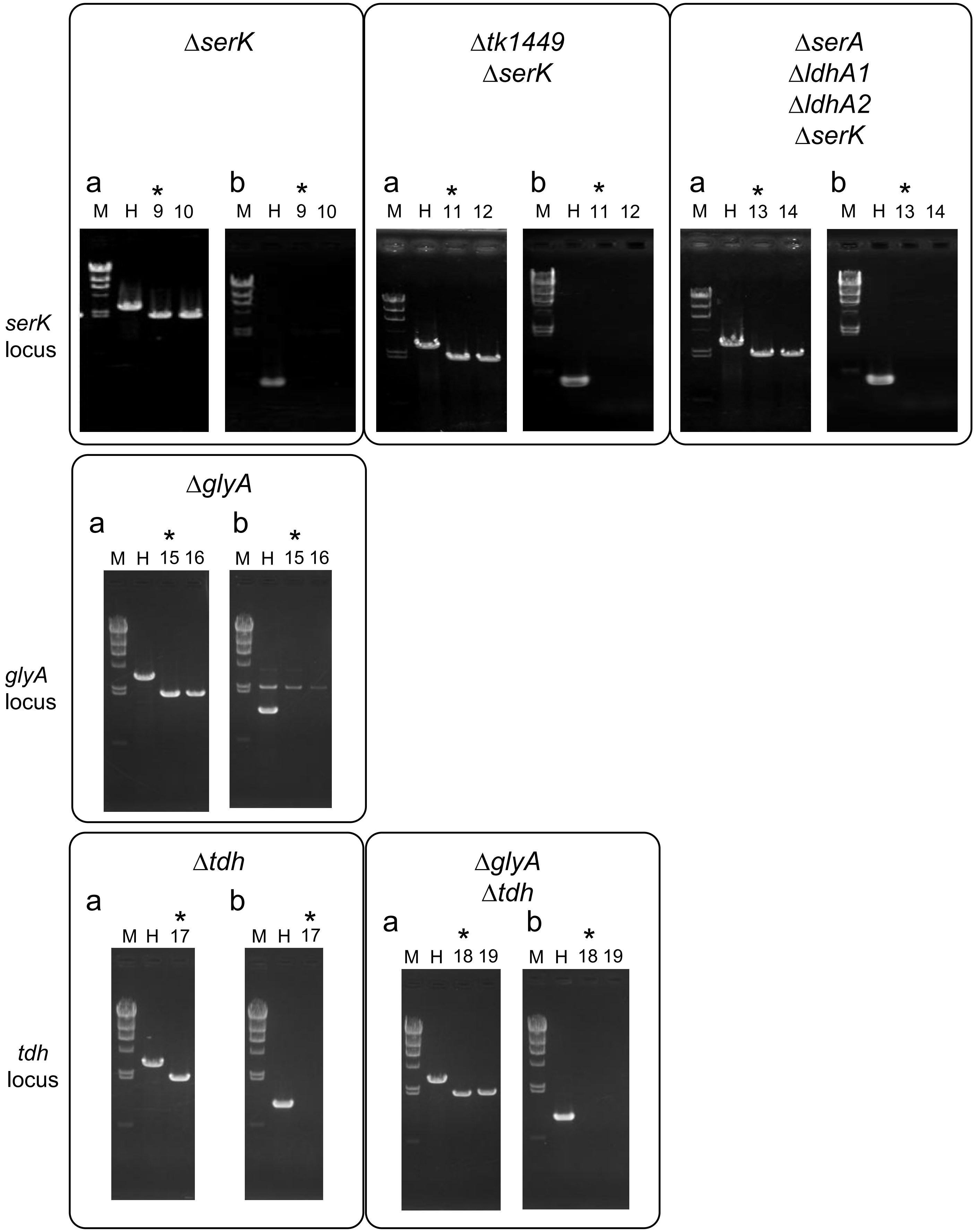
ΔserK
Δtk1449
ΔserK
ΔserA
ΔldhA1
ΔldhA2
ΔserK
a
b
M H * 9 10
M H * 11 12
M H * 13 14
serK locus
ΔglyA
M H * 15 16
glyA locus
Δtdh
M H * 17
ΔglyA
Δtdh
M H * 18 19
tdh locus

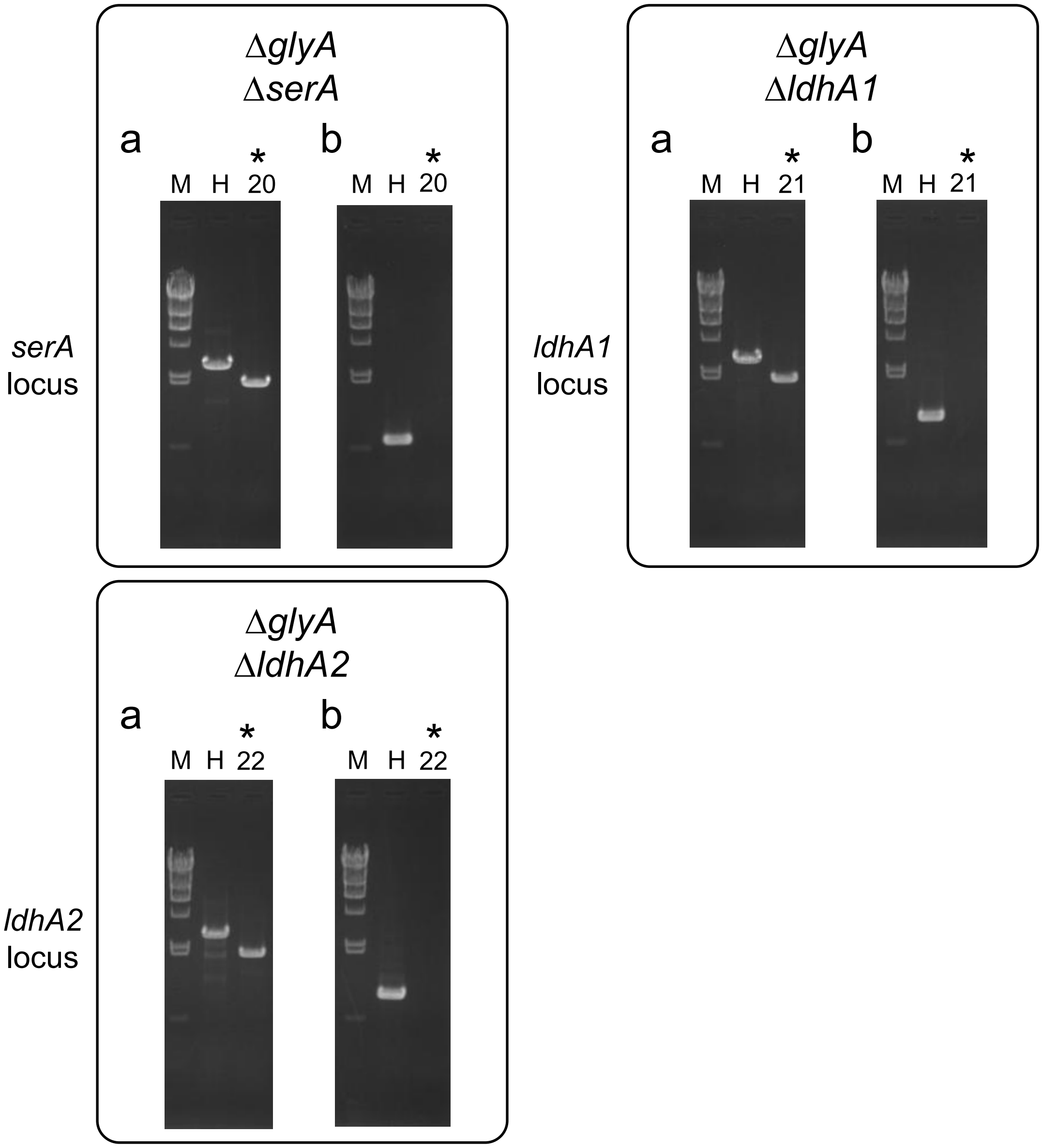

Supplementary Figure 3. PCR analysis of gene disruption strains. Genotypes of disruptants were analyzed by PCR using the primer sets annealing outside of the 5'- and 3'-homologous regions for homologous recombination **(a)** and within the target genes **(b)**. When analyzing the *cysK* disruptants (Δ*pyrF* Δ*cysK*::*pyrF*), we utilized primers annealing outside of the 5'-homologous regions and within the marker *pyrF* gene **(c)**. Abbreviations; M: Marker, W: *T. kodakarensis* KOD1 wild-type strain, H: *T. kodakarensis* KU216 host strain, 1: Δ*tk1449*-2-1-2*, 2: Δ*tk1449*-2-1-15, 3: Δ*serA*Δ*ldhA1*Δ*ldhA2*-1*, 4: Δ*serA*Δ*ldhA1*Δ*ldhA2*-27, 5: Δ*serA*Δ*ldhA1*Δ*ldhA2*Δ*tk1449*-2*, 6: Δ*serA*Δ*ldhA1*Δ*ldhA2*Δ*tk1449*-6, 7: Δ*cysK*-3*, 8: Δ*cysK*-7, 9: Δ*serK*-1*, 10: Δ*serK*-2, 11: Δ*tk1449*Δ*serK*-1*, 12: Δ*tk1449*Δ*serK*-2, 13: Δ*serA*Δ*ldhA1*Δ*ldhA2*Δ*serK*-1*, and 14: Δ*serA*Δ*ldhA1*Δ*ldhA2*Δ*serK*-2, 15: Δ*glyA*-61*, 16: Δ*glyA*-63, 17: Δ*tdh*-16*, 18: Δ*glyA*Δ*tdh*-22*, 19: Δ*glyA*Δ*tdh*-27, 20: Δ*glyA*Δ*serA*-1*, 21: Δ*glyA*Δ*ldhA1*-3* and 22: Δ*glyA*Δ*ldhA2*-1*. λDNA digested with HindIII was used as a DNA marker (including 23.1, 9.4, 6.6, 4.4, 2.3, 2.0, and 0.6 kbp-fragments). The strains indicated by an asterisk were used in further growth experiments.

Supplementary Figure 4

```
Group A
Ec-CysK-A          (193)  KGKTDLISVAVEPTDSPVIAQALAGEEIKPGPHKIQGIGAG---FIPANLDLK
St-CysK-A          (193)  KGKTDLITVAVEPTDSPVIAQALAGEEIKPGPHKIQGIGAG---FIPGNLDLK
Eh-CysK            (208)  KKGIKII--AVEPEESAVLEGK------AKGPHGIQGIGAG---FIPDIYKKE
Lm-CysK            (207)  GSHARIV--AVEPMESPVLSGG------KPGAHKIQGIGPG---FVPDVLDRS
At-CysK            (197)  NANVKLY--GVEPVESAILSGG------KPGPHKIQGIGAG---FIPSVLNVD
At-CysK-chlor      (267)  KPELKVI--GVEPTESAILSGG------KPGPHKIQGIGAG---FVPKNLDLA
At-CysK-mitochon   (305)  NPKTQVI--GVEPTESDILSGG------KPGPHKIQGIGAG---FIPKNLDQK
Mt-CysK            (194)  RPSFKAI--AVEPAESPVLSGG------SPGPHRIQGIGAG---FVPDVLQLE

Group B
Ec-CysK-B          (190)  SKPVTIV--GLQPEE----------------GSSIPGIRRWPAEYLPGIFNAS
St-CysK-B          (190)  EKPVTIV--GLQPEE----------------GSSIPGIRRWPAEYMPGIFNAS
Gs-CysK            (196)  KPSVRIV--GVEPRL----------------GHKVQGLKNMQEAIVPPIYHPE
Tv-CysK            (194)  NPEIKII--EAQPTK----------------GHYIQGLKSMEEAIVPAIYQAD
Tk-CysK            (169)  YDTKVI---GVVPAK----------------NEKIPGIKRLETRPKW--FFDA
Pf-CysK            (169)  YGTKII---GVVPAE----------------GEKIPGIKRIETKPKW--FFQV
Dm-CysK            (285)  YGRVKTI--GVQPAQ----------------GSSIPGLRRIETGVKW--LHSV
Ap-CysK            (277)  DPSIRAV--LVQPAQ----------------GDSIPGIRRVETGMLW-INMLD
                                                              ooooooo
                                                              ▲
```

Supplementary Figure 4. Sequence alignment of CysKs from various organisms.

The amino acid residues displayed are a portion of a loop region between the β8 and β9 strands in cysteine synthase (CysK). This sequence alignment is based on the alignment constructed by G. D. Westrop et al.[3]. Amino acid sequences of CysK from *T. kodakarensis* (*Tk*-CysK), *Methanosarcina thermophila* (*Mt*-CysK) and *Desulfurococcus mucosus* (*Dm*-CysK) were added to the already-constructed alignment. Abbreviations; *Ec*: *Escherichia coli*, *St*: *Salmonella typhimurium*, *Eh*: *Entamoeba histolytica*, *Lm*: *Leishmania major*, *At*: *Arabidopsis thaliana*, *Mt*: *Methanosarcina thermophila*, *Gs*: *Geobacter sulfurreducens*, *Tv*: *Trichomonas vaginalis*, *Tk*: *Thermococcus kodakarensis*, *Pf*: *Pyrococcus furiosus*, *Dm*: *Desulfurococcus mucosus, Ap*: *Aeropyrum pernix*, CysK-mitochon: mitochondrial CysK, CysK-chlor: CysK in chloroplast. Based on the primary sequence, CysKs are classified into two groups, A and B. Highly conserved residues throughout all CysKs are indicated by white characters in red (similar residues are in pink background). Those conserved only in type A CysKs are indicated in blue background (similar residues, light blue). Those conserved

only in type B CysKs are indicated in black background (similar residues, gray). Type A CysKs are distributed in Bacteria, protozoa and plants, while type B CysKs are found in Bacteria, protozoa and Archaea. *Tk*-CysK and *Dm*-CysK are members of the type B CysK. In general, type A CysKs utilize *O*-acetylserine and hydrogen sulfide to synthesize Cys. On the other hand, bacterial type B CysKs can use thiosulfate as a sulfur donor in addition to hydrogen sulfide. In the case of type B CysK from *E. coli* [Ec-CysK-B], the loop formed by 7 amino acid residues (Arg_{210}-Tyr_{216}), indicated by open circles, is considered to enlarge the substrate pocket and allow the recognition of thiosulfate. This region is also found in the CysK from the hyperthermophilic archaeon *A. pernix* (*Ap*-CysK), which utilizes *O*-phosphoserine (Sep) rather than *O*-acetylserine as a substrate. Positively-charged residues in this region, indicated with a closed arrowhead, (Arg_{210} of *Ec*-CysK-B, Lys_{214} of *Tv*-CysK[3] and Arg_{297} of *Ap*-CysK[10]) are suggested to recognize the negatively-charged thiosulfate or Sep[3, 10]. The extra loop and positively-charged residue are present in *Tk*-CysK, raising the possibility that Cys is synthesized from Sep, which is catalyzed by CysK in *T. kodakarensis*.

Supplementary Figure 5

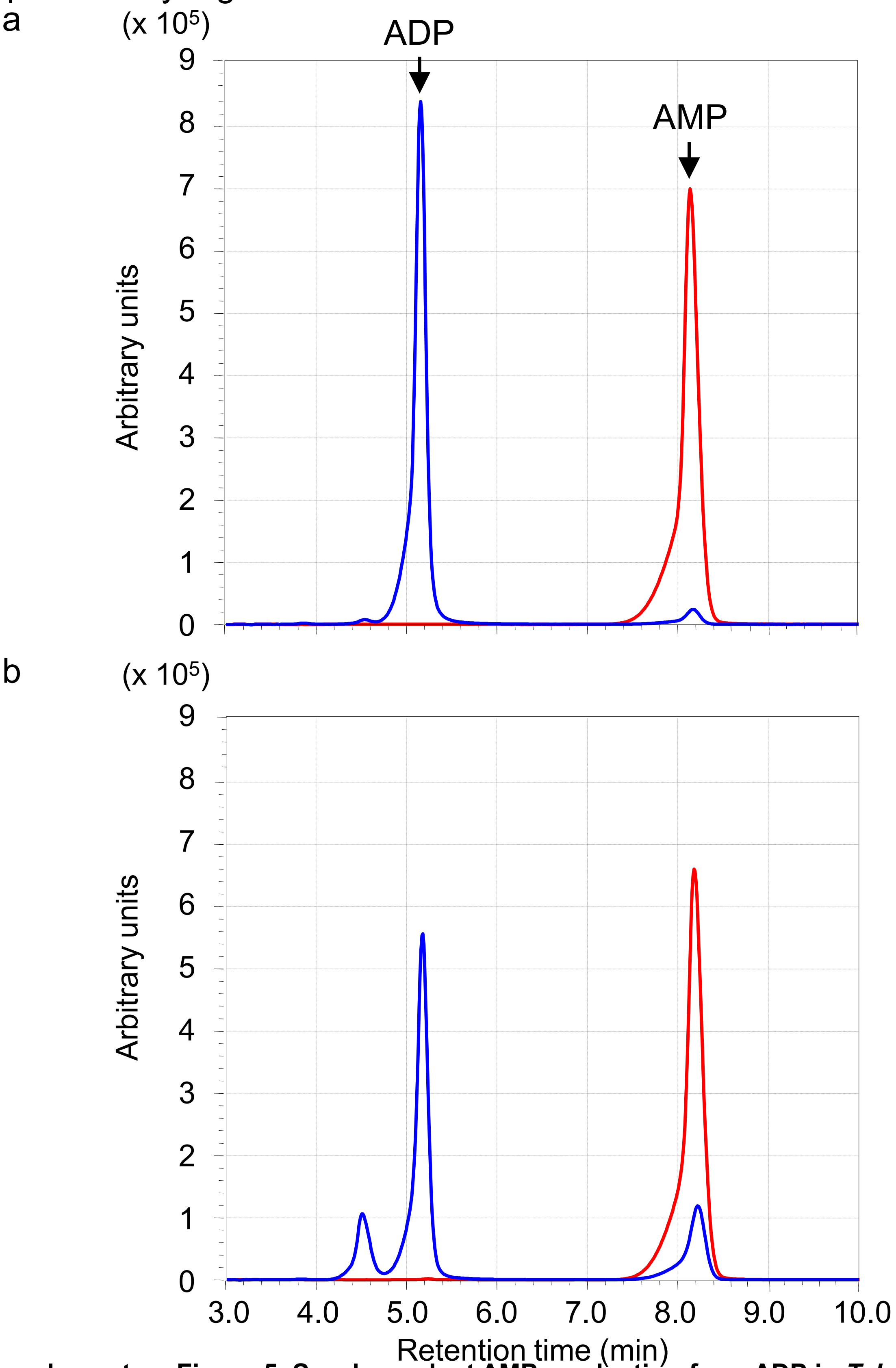

Supplementary Figure 5. Ser-dependent AMP production from ADP in *T. kodakarensis* cell-free extracts. ADP and AMP standards **(a)** and reaction products **(b)** were examined by HPLC. (a) Blue line,1 mM standard ADP; and red line, 1 mM standard AMP. (b) Blue line, reaction product without Ser; red line, reaction product with Ser.

Supplementary Figure 6

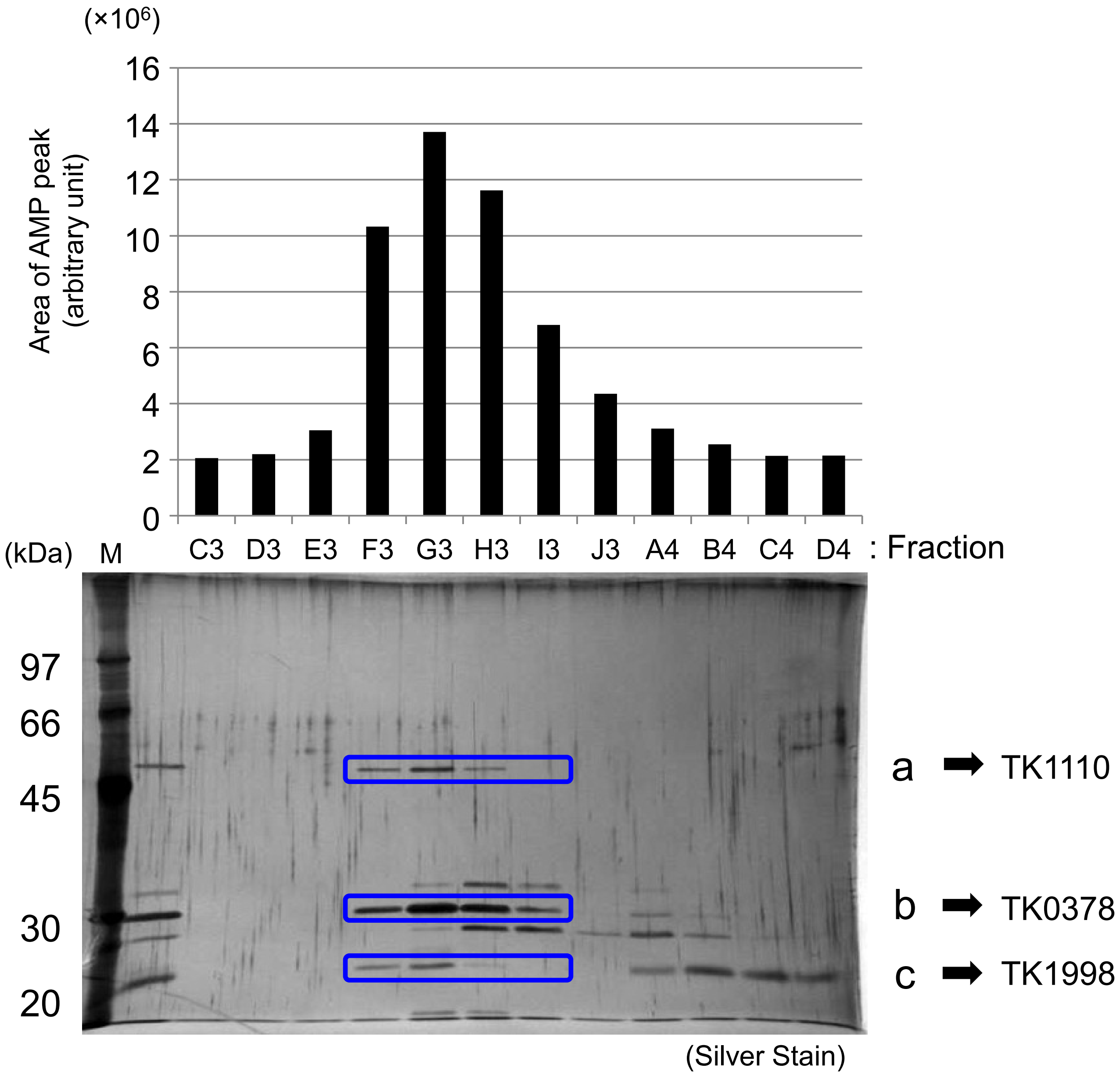

Supplementary Figure 6. Identification of three SerK candidates by activity measurement, SDS-PAGE and LC-MS analyses. Proteins with Ser-dependent AMP generating activity from ADP were purified from cell-free extracts of *T. kodakarensis* as described in the Methods section. After gel filtration chromatography, each fraction was analyzed by AMP-forming activity measurements (upper panel) and SDS-PAGE (lower panel). Three proteins whose band intensities correlated with AMP-forming activity were identified, and are indicated by blue boxes (a, b, c). These were analyzed by LC-MS analysis and identified as TK1110, TK0378 and TK1998 proteins. M represents molecular weight marker.

Supplementary Figure 7

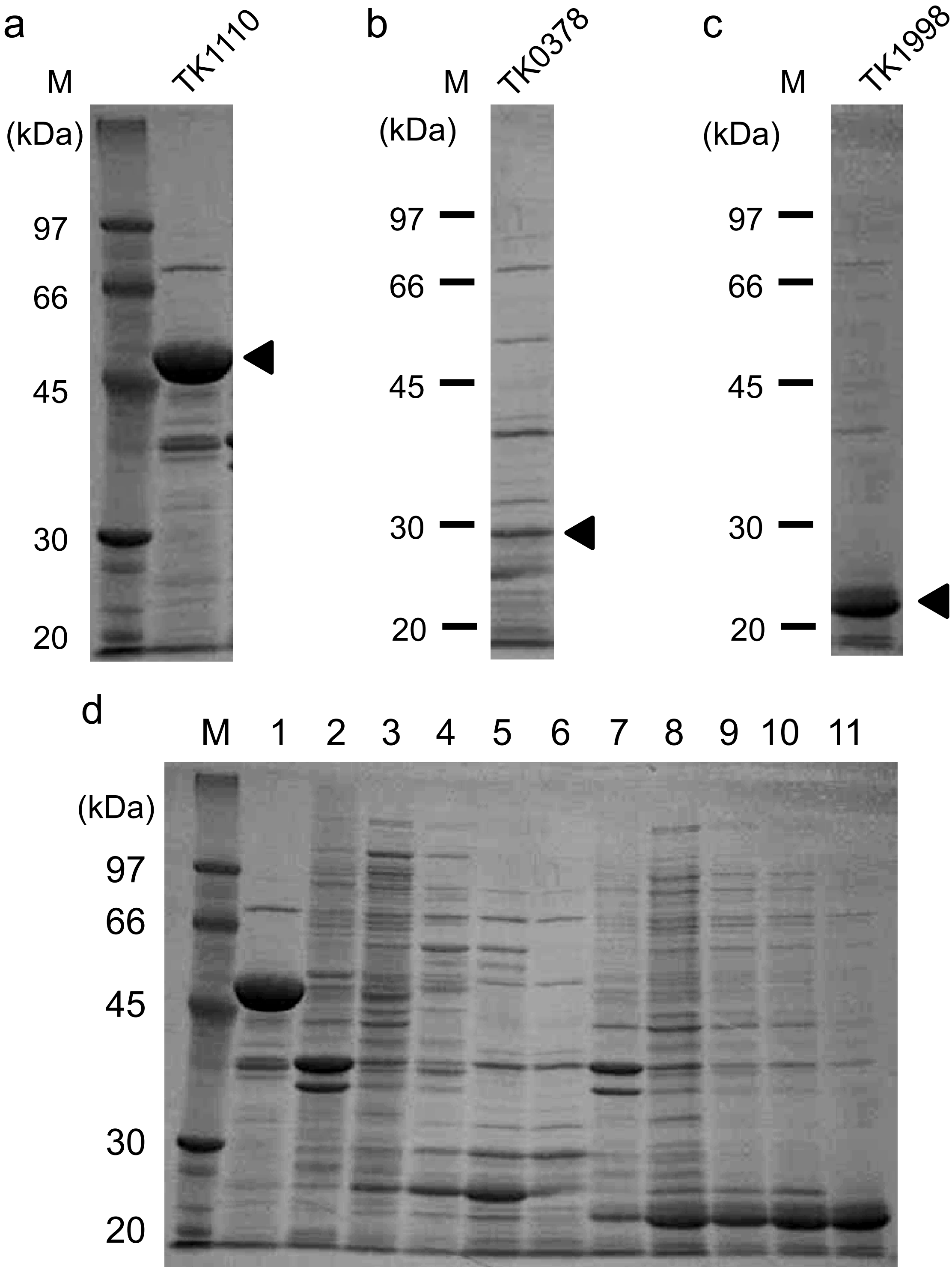

Supplementary Figure 7. SDS-PAGE analysis of the recombinant proteins of three SerK candidates. The three recombinant proteins, TK1110 **(a)**, TK0378 **(b)** and TK1998 **(c)**, produced in *E. coli* and partially purified with heat treatment at 80˚C were analyzed by SDS-PAGE electrophoresis and stained with Coomassie Brilliant Blue. M represents molecular weight marker. The original gel from which (a-c) were cropped is also displayed **(d)**. Lane 1:

supernatant of cell extracts including TK1110 after 80˚C heat treatment, lane 2: precipitate of cell extracts including TK0378 after sonication, lane 3: supernatant of cell extracts including TK0378 after sonication, lane 4: supernatant of cell extracts including TK0378 after 60˚C heat treatment, lane 5: supernatant of cell extracts including TK0378 after 70˚C heat treatment, lane 6: supernatant of cell extracts including TK0378 after 80˚C heat treatment, lane 7: precipitate of cell extracts including TK1998 after sonication, lane 8: supernatant of cell extracts including TK1998 after sonication, lane 9: supernatant of cell extracts including TK1998 after 60˚C heat treatment, lane 10: supernatant of cell extracts including TK1998 after 70˚C heat treatment, lane 11: supernatant of cell extracts including TK1998 after 80˚C heat treatment.

Supplementary Figure 8

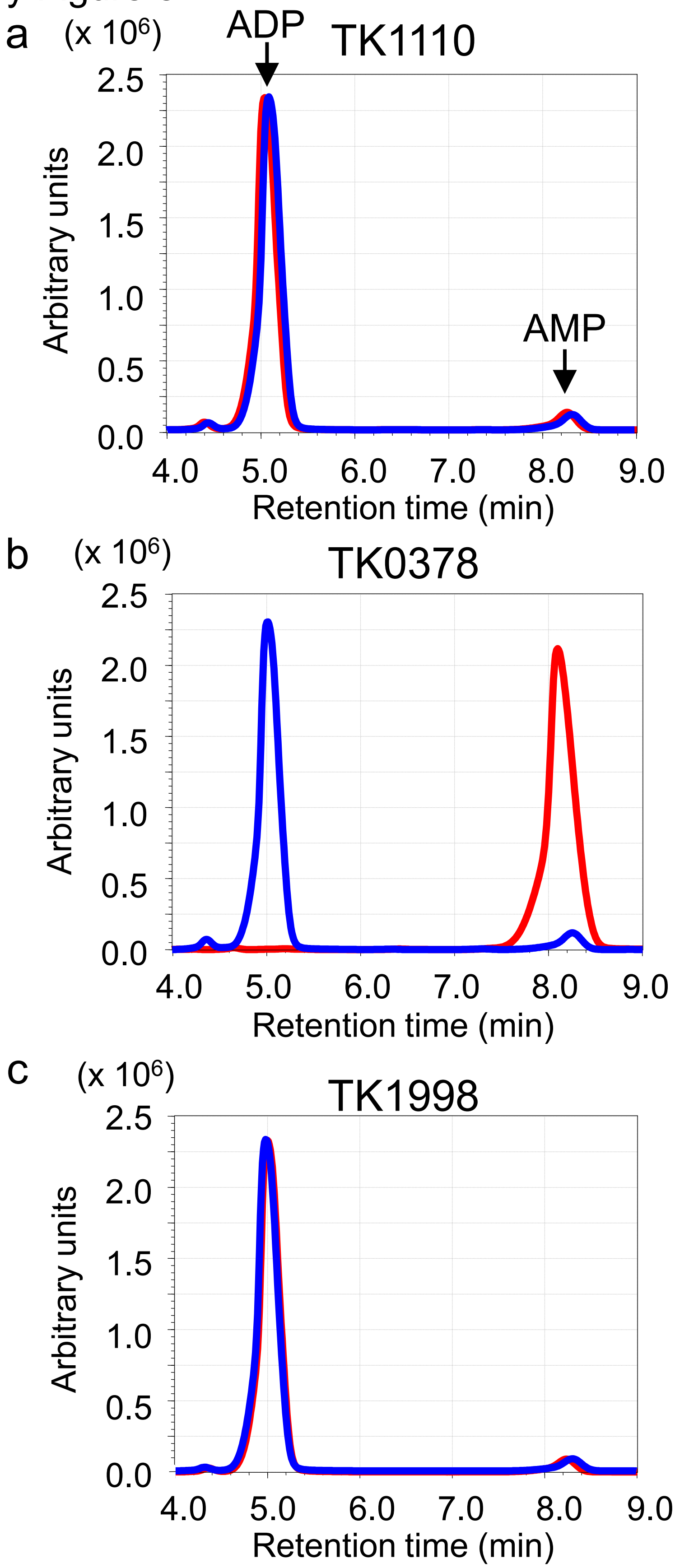

Supplementary Figure 8. ADP-dependent serine kinase activity of the recombinant proteins of TK1110 (a), TK0378 (b) and TK1998 (c). Blue line, reaction product without Ser; and red line, reaction product with Ser.

Supplementary Figure 9

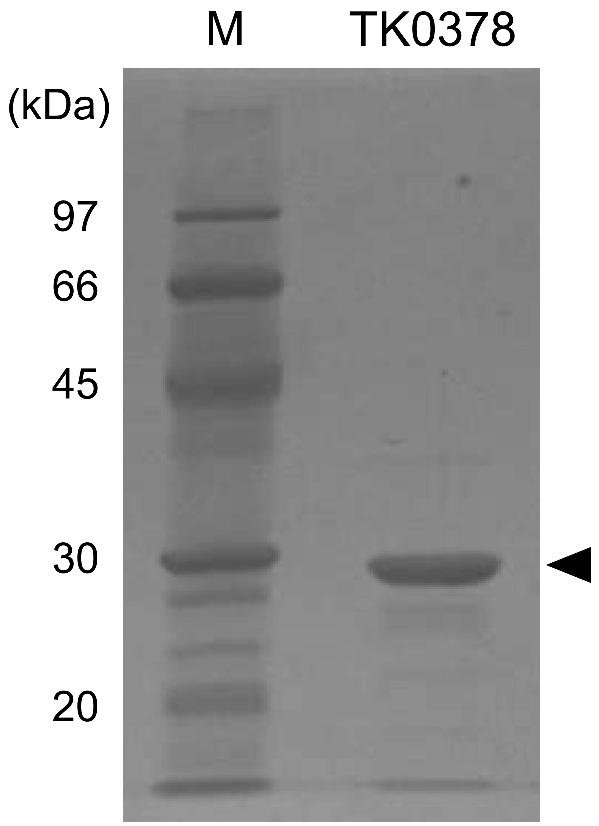

Supplementary Figure 9. Purified recombinant TK0378 protein. Purified recombinant TK0378 protein was analyzed by SDS-PAGE electrophoresis and stained with Coomassie Brilliant Blue. M represents molecular weight marker.

Supplementary Figure 10

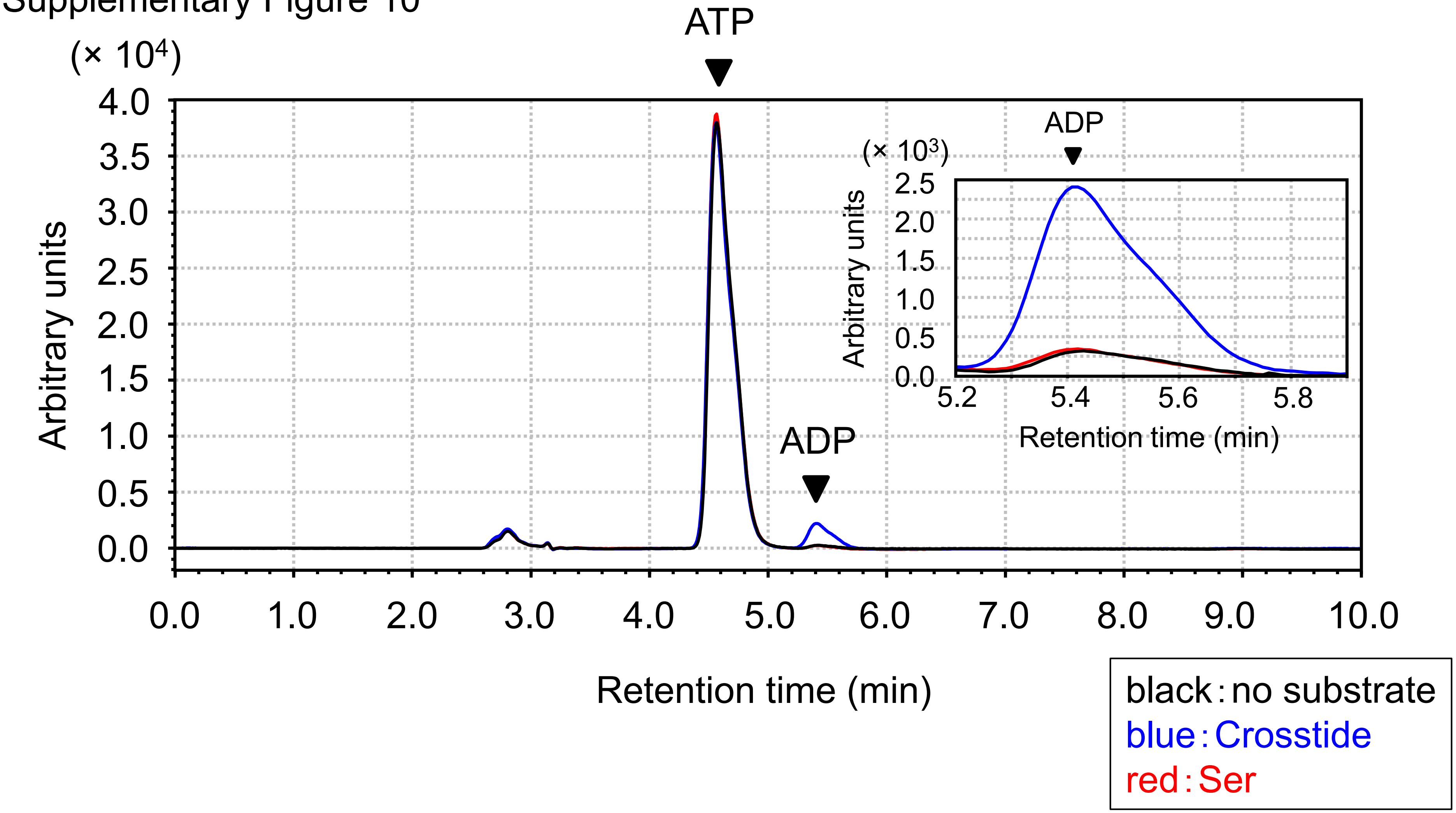

Supplementary Figure 10. Phosphorylating activities of RAC-alpha serine/threonine-protein kinase (Akt1) toward Ser residue in Crosstide and free Ser. "Crosstide" is an eleven-residue peptide with two Ser residues (Gly-Arg-Pro-Arg-Thr-Ser-Ser-Phe-Ala-Glu-Gly) analogous to glycogen synthase kinase-3 (GSK-3). GSK-3 and Crosstide are known to be substrates for Akt1, human Ser protein kinase. Black line, no phosphate acceptor; blue line, 100 μM Crosstide; red line, Ser. The upper-right inset displays the peaks of ADP.

Supplementary Figure 11

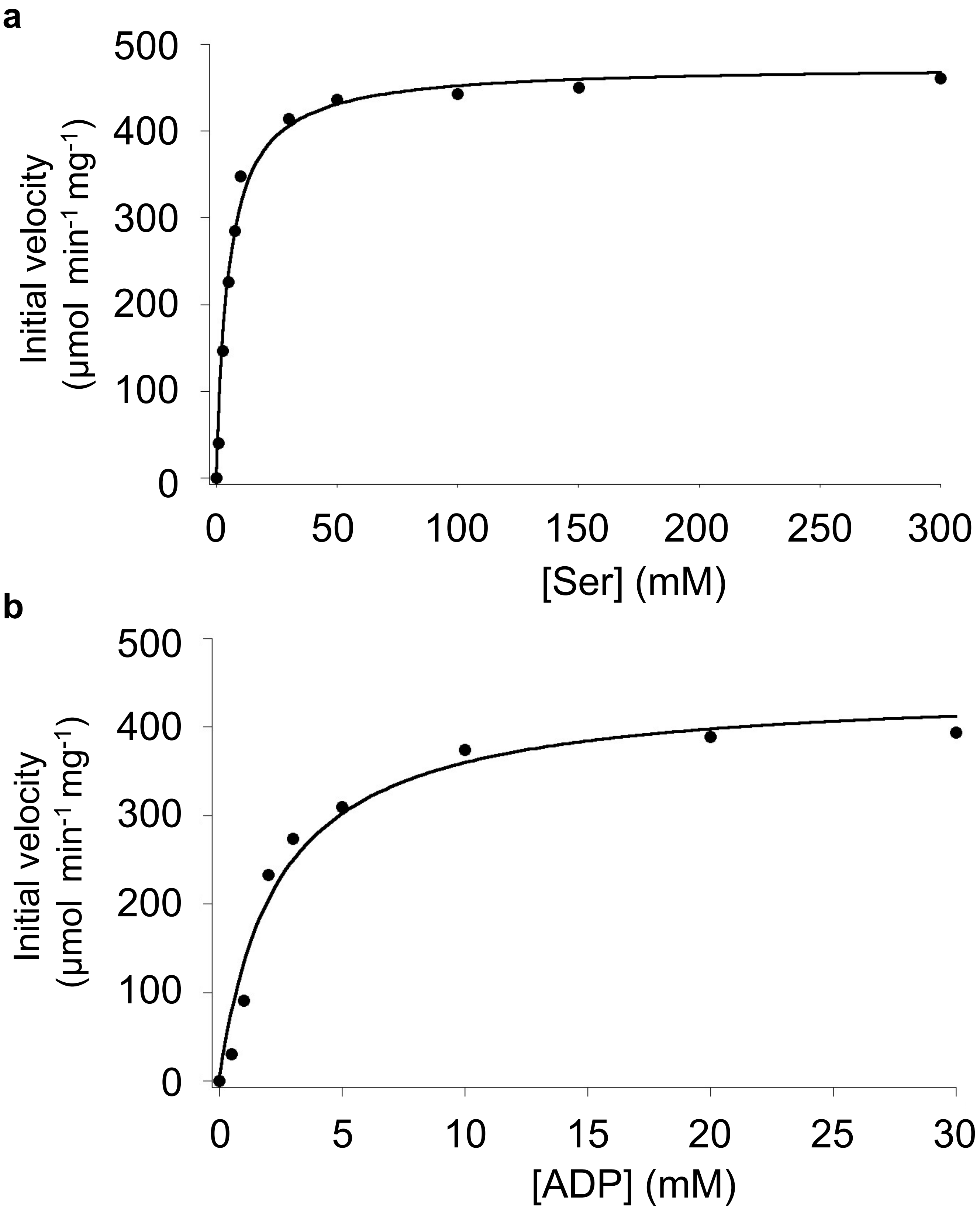

Supplementary Figure 11. Kinetic analysis of TK0378 protein. Initial velocities of the Ser kinase reaction in the presence of varying concentrations of Ser with 20 mM ADP **(a)** and in the presence of varying concentrations of ADP with 50 mM Ser **(b)**. All reactions were carried out at 85ºC.

Supplementary Figure 12

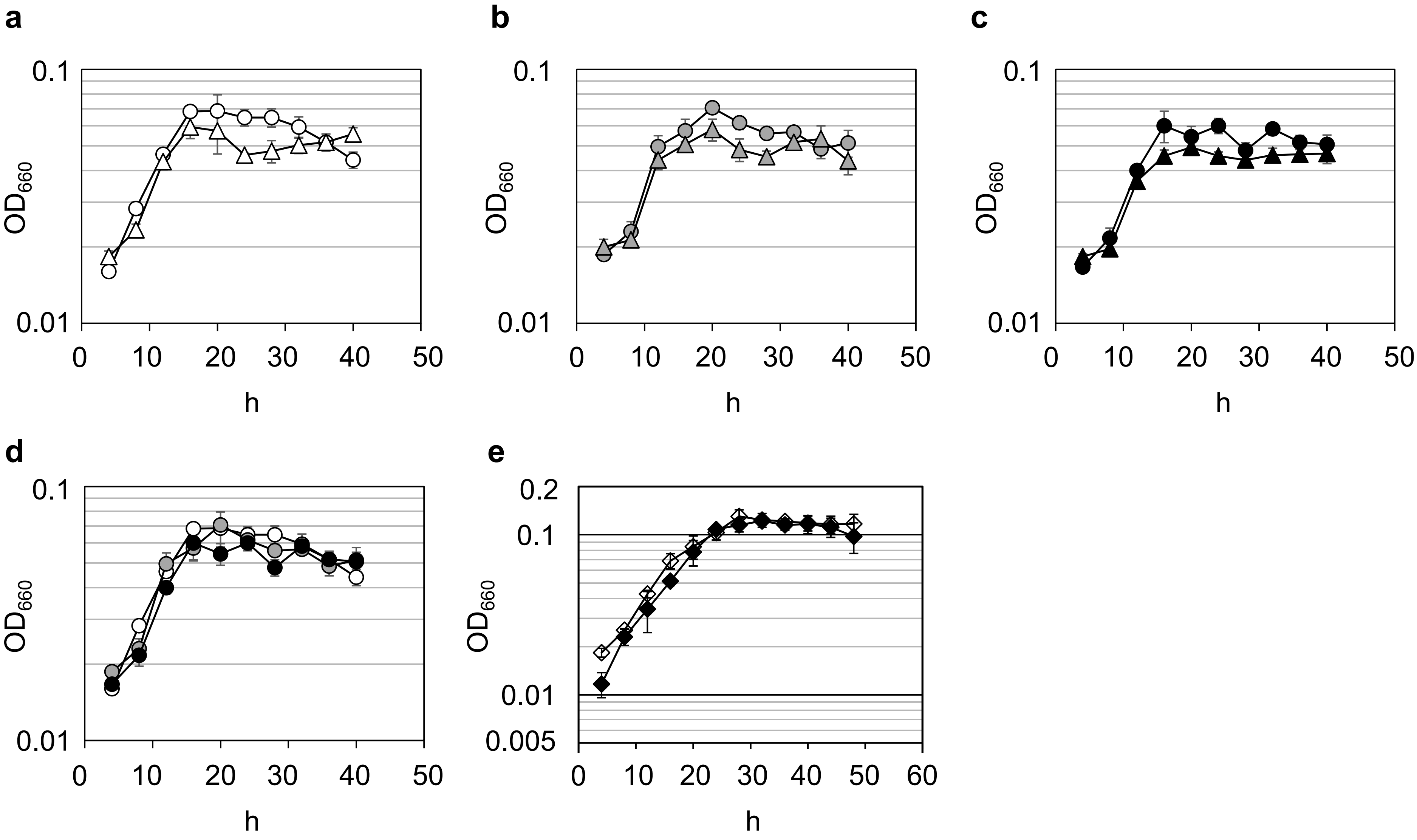

Supplementary Figure 12. Growth properties of the host strain KU216 and the Δ*serK* strain in the presence of various concentrations of Ser. *T. kodakarensis* KU216 and Δ*serK* strains were cultivated in the presence of various concentrations (0.71 mM **(a)**, 1.42 mM **(b)** and 2.13 mM **(c)**) of Ser. The growth curves of the KU216 host strain in **(a)**-**(c)** were merged in **(d)**. The growth curves of the KU216 host strain grown with (0.71 mM) or without Ser, shown in Figure 5a,b, were merged in **(e)**. Error bars indicate the standard deviations of three independent culture experiments.

Supplementary Figure 13

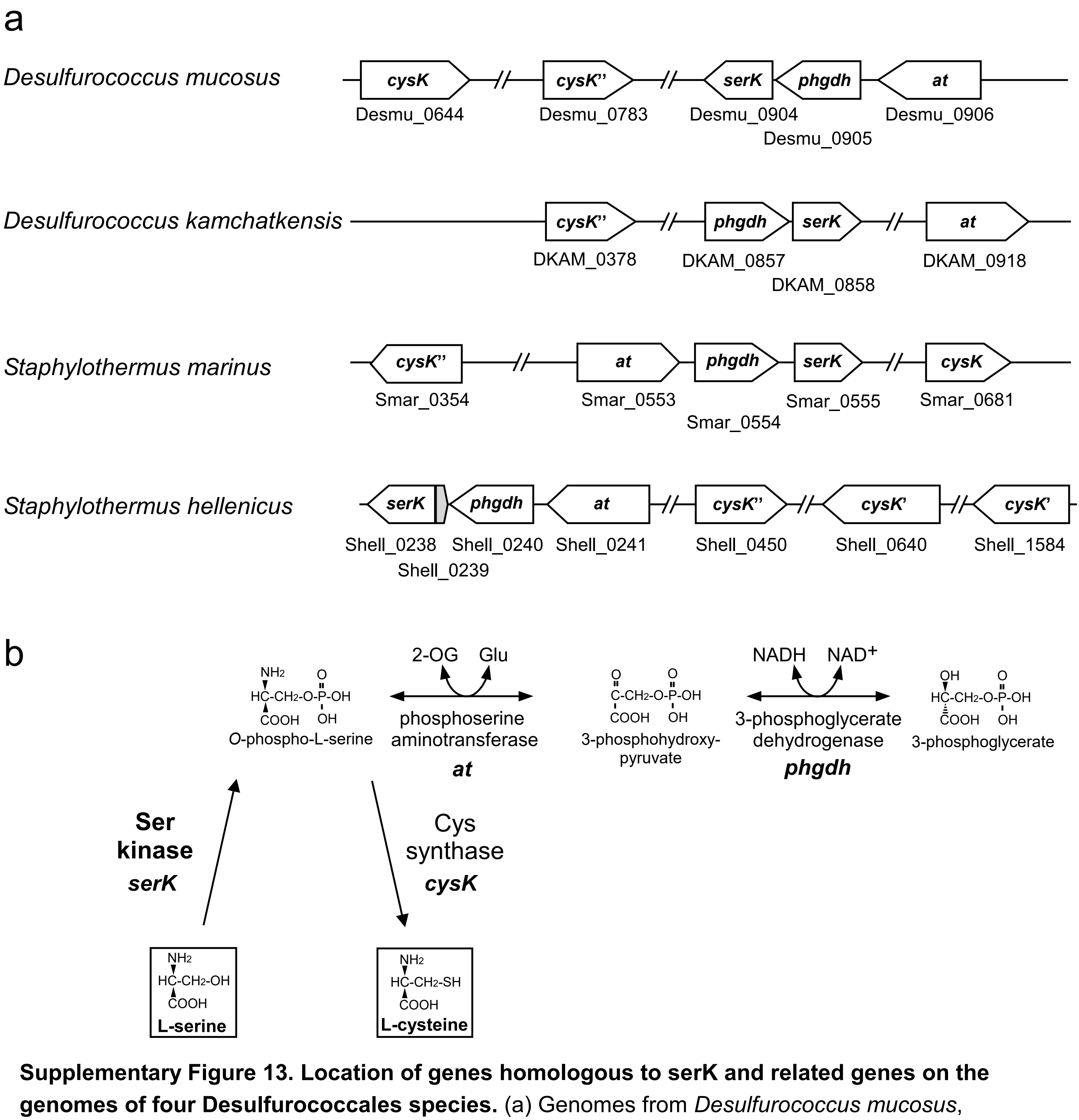

Supplementary Figure 13. Location of genes homologous to serK and related genes on the genomes of four Desulfurococcales species. (a) Genomes from *Desulfurococcus mucosus*, *Desulfurococcus kamchatkensis*, *Staphylothermus marinus* and *Staphylothermus hellenicus* are shown. Gene abbreviations; *serK*: *Tk-serK* homologs, *phgdh*: genes annotated as phosphoglycerate dehydrogenase or D-isomer specific 2-hydroxyacid dehydrogenase, *at*: genes annotated as aminotransferase, *cysK*: genes with high similarity with *Tk-cysK* that possess the insertion sequence found in CysK proteins utilizing Sep (see Supplementary Fig. 4), *cysK*': genes with high similarity with *Tk-cysK* but which lack the insertion sequence, *cysK*'': genes with moderate similarity with *Tk-cysK* and lacking the insertion sequence. The gray box indicates a putative hypothetical protein.
(b) Predicted metabolic pathways involving SerK homologs in Desulfurococcales based on gene location and presence of CysK. The gene clustering of *serK*, *at* and *phgdh* suggest a metabolism from Ser to 3-phosphoglycerate in all species. The presence of CysK suggests additional conversion from Ser to Cys in *D. mucosus* and *S. marinus*. Abbreviation; 2-OG: 2-oxoglutarate.

Supplementary Table 1. Strains and plasmids used in this study

Strain or plasmid	Relevant characteristic(s)	Source or reference
Strains		
Escherichia coli		
DH5α	*supE44* Δ*lacU169* (Φ80 *lacZ* Δ*M15*) *hsdR17 recA1 endA1 gyrA96 thi-1 relAI*	Stratagene (La Jolla, CA)
BL21-CodonPlus(DE3)-RIL	*E. coli* B F^- *ompT hsdS*(r_B^- m_B^-) dcm^+ Tet^r *gal* λ(DE3) *endA* Hte (*argU ileY leuW* Cam^r)	Stratagene
Thermococcus kodakarensis		
KOD1	Wild-type	11
KU216	KOD1 Δ*pyrF*	8
Δ*tk1449*-2-1-2*	KU216 Δ*tk1449* (annotated as *metC*)	This study
Δ*tk1449*-2-1-15	KU216 Δ*tk1449* (annotated as *metC*)	This study
Δ*serA*Δ*ldhA1*Δ*ldhA2*-1*	KU216 Δ*serA* Δ*ldhA1* Δ*ldhA2*	This study
Δ*serA*Δ*ldhA1*Δ*ldhA2*-27	KU216 Δ*serA* Δ*ldhA1* Δ*ldhA2*	This study
Δ*serA*Δ*ldhA1*Δ*ldhA2*Δ*tk1449*-2*	Δ*serA*Δ*ldhA1*Δ*ldhA2*-1 Δ*tk1449*	This study
Δ*serA*Δ*ldhA1*Δ*ldhA2*Δ*tk1449*-6	Δ*serA*Δ*ldhA1*Δ*ldhA2*-1 Δ*tk1449*	This study
Δ*cysK*-3*	KU216 Δ*cysK*::*pyrF*	This study
Δ*cysK*-7	KU216 Δ*cysK*::*pyrF*	This study
Δ*serK*-1*	KU216 Δ*serK*	This study
Δ*serK*-2	KU216 Δ*serK*	This study
Δ*tk1449*Δ*serK*-1*	Δ*tk1449*-2-1-2 Δ*serK*	This study
Δ*tk1449*Δ*serK*-2	Δ*tk1449*-2-1-2 Δ*serK*	This study
Δ*serA*Δ*ldhA1*Δ*ldhA2*Δ*serK*-1*	Δ*serA*Δ*ldhA1*Δ*ldhA2*-1 Δ*serK*	This study
Δ*serA*Δ*ldhA1*Δ*ldhA2*Δ*serK*-2	Δ*serA*Δ*ldhA1*Δ*ldhA2*-1 Δ*serK*	This study
Δ*glyA*-61*	KU216 Δ*glyA*	This study
Δ*glyA*-63	KU216 Δ*glyA*	This study
Δ*tdh*-16*	KU216 Δ*tdh*	This study

Δ*glyA*Δ*tdh*-22*	Δ*glyA*-61 Δ*tdh*	This study
Δ*glyA*Δ*tdh*-27	Δ*glyA*-61 Δ*tdh*	This study
Δ*glyA*Δ*serA*-1*	Δ*glyA*-61 Δ*serA*	This study
Δ*glyA*Δ*ldhA1*-3*	Δ*glyA*-61 Δ*ldhA1*	This study
Δ*glyA*Δ*ldhA2*-1*	Δ*glyA*-61 Δ*ldhA2*	This study
Plasmids		
pUC118	Amp^r general cloning vector	Takara (Kyoto, Japan)
pUD2	pUC118 derivative, *pyrF* marker cassette (P_{pyrF}::*pyrF*)	8
pUD3	pUC118 derivative; *pyrF* marker cassette (P_{pyrF}::*pyrF*)	12
pUDTK1449	pUD3 derivative; *metC* disruption vector	This study
pUDTK1966	pUD3 derivative; *serA* disruption vector	This study
pUDTK0551	pUD3 derivative; *ldhA1* disruption vector	This study
pUDTK0683	pUD3 derivative; *ldhA2* disruption vector	This study
pUDTK1687	pUC118 derivative; Δ*cysK*::*pyrF*: *cysK* disruption vector	This study
pUDTK0378	pUD3 derivative; *serK* disruption vector	This study
pUDTK0528	pUD3 derivative; *glyA* disruption vector	This study
pUDTK0916	pUD3 derivative; *tdh* disruption vector	This study
pET-21a(+)	Amp^r general expression vector	Novagen (Madison, Wis.)
pET-TK0378	pET-21a(+) derivative; *tk0378*	This study
pET-TK1110	pET-21a(+) derivative; *tk1110*	This study
pET-TK1998	pET-21a(+) derivative; *tk1998*	This study

T. kodakarensis mutant strains with asterisk were examined in growth experiments.

Supplementary Table 2. List of proteins detected in LC-MS analysis for identification of ADP-dependent Ser kinase

Band a

TK number	Description	Score	Coverage
TK1110	ADP-dependent glucokinase	164.51	91.83%
TK0378	Chromosome partitioning protein ParB homolog	8.61	37.60%
TK0308	Translation elongation factor EF-1, alpha subunit	3.76	21.96%
TK1597	V-type ATP synthase subunit I	2.58	2.71%
TK0544	Molybdenum cofactor biosynthesis protein B	2.37	14.71%

Band b

TK number	Description	Score	Coverage
TK0378	Chromosome partitioning protein ParB homolog	174.51	95.04%
TK0878	Translation initiation factor 5A	6.61	20.59%
TK0544	Molybdenum cofactor biosynthesis protein B	2.74	18.24%
TK0560	DNA/RNA-binding protein Alba	2.20	25.27%
TK2255	Bifunctional phosphatase/dolichol-phosphate glucotransferase	1.69	5.91%

Band c

TK number	Description	Score	Coverage
TK1998	Putative uncharacterized protein	84.72	89.16%
TK0378	Chromosome partitioning protein ParB homolog	40.46	67.77%
TK1110	ADP-dependent glucokinase	9.11	7.51%
TK0878	Translation initiation factor 5A	4.56	20.59%
TK0544	Molybdenum cofactor biosynthesis protein B	2.31	11.76%

Supplementary Table 3. List of proteins possessing homology with *Tk*-SerK

		Organism	K number score	E-value
1	tko:TK0378 chromosome partitioning protein ParB-like protein	*Thermococcus kodakarensis*	480	2.00E−170
2	tnu:BD01_0459 putative transcriptional regulators	*Thermococcus nautili*	462	2.00E−163
3	the:GQS_01905 chromosome partitioning protein ParB-like prc	*Thermococcus* sp. 4557	459	4.00E−162
4	tha:TAM4_25 chromosome partitioning protein ParB-like protei	*Thermococcus* sp. AM4	458	8.00E−162
5	tga:TGAM_1907 parB-like nuclease	*Thermococcus gammatolerans*	458	1.00E−161
6	thm:CL1_0055 chromosome partitioning protein ParB-like prot	*Thermococcus cleftensis*	457	3.00E−161
7	teu:TEU_09925 chromosome partitioning protein ParB	*Thermococcus eurythermalis*	456	5.00E−161
8	ton:TON_0620 chromosome partitioning protein	*Thermococcus onnurineus*	444	3.00E−156
9	pyn:PNA2_1158 hypothetical protein	*Pyrococcus* sp. NA2	436	9.00E−153
10	pya:PYCH_19090 parB-like nuclease	*Pyrococcus yayanosii*	426	6.00E−149
11	tsi:TSIB_0895 ParB-like nuclease	*Thermococcus sibiricus*	426	6.00E−149
12	ths:TES1_0806 chromosome partitioning protein ParB-like pro	*Thermococcus* sp. ES1	420	1.00E−146
13	tba:TERMP_00746 hypothetical protein	*Thermococcus barophilus*	417	2.00E−145
14	ppac:PAP_07195 chromosome partitioning protein ParB	*Palaeococcus pacificus*	417	2.00E−145
15	pab:PAB1362 hypothetical protein	*Pyrococcus abyssi*	416	3.00E−145
16	tlt:OCC_10970 chromosome partitioning protein ParB	*Thermococcus litoralis*	417	1.00E−144
17	pho:PH0530 hypothetical protein	*Pyrococcus horikoshii*	412	1.00E−143
18	pys:Py04_0575 parB-like nuclease	*Pyrococcus* sp. ST04	407	2.00E−141
19	pfu:PF0380 hypothetical protein	*Pyrococcus furiosus* DSM 3638	402	1.00E−139
20	pfi:PFC_00865 hypothetical protein	*Pyrococcus furiosus* COM1	402	2.00E−139
21	smr:Smar_0555 nuclease	*Staphylothermus marinus*	225	7.00E−70
22	shc:Shell_0238 ParB domain-containing protein nuclease	*Staphylothermus hellenicus*	223	4.00E−69
23	dmu:Desmu_0904 ParB domain-containing protein nuclease	*Desulfurococcus mucosus*	204	9.00E−62
24	dka:DKAM_0858 ParB domain-containing protein nuclease	*Desulfurococcus kamchatkensis*	198	2.00E−59
25	dfd:Desfe_0944 ParB domain-containing protein nuclease	*Desulfurococcus fermentans*	198	2.00E−59
26	hbu:Hbut_1584 transcriptional regulator	*Hyperthermus butylicus*	81.3	3.00E−16
27	thg:TCELL_0807 ParB domain-containing protein nuclease	*Thermogladius cellulolyticus*	80.5	2.00E−15
28	tag:Tagg_1147 ParB domain-containing protein nuclease	*Thermosphaera aggregans*	77	4.00E−14
29	asc:ASAC_0963 hypothetical protein	*Acidilobus saccharovorans*	63.2	1.00E−09
30	pfm:Pyrfu_0683 ParB domain containing protein nuclease	*Pyrolobus fumarii*	62.8	3.00E−09
31	acj:ACAM_0517 predicted transcriptional regulator	*Aeropyrum camini*	58.5	4.00E−08
32	thb:N186_05155 hypothetical protein	*Thermofilum* sp. 1910b	56.6	2.00E−07
33	tpe:Tpen_1066 nuclease	*Thermofilum pendens*	56.2	2.00E−07
34	iag:Igag_1782 ParB domain-containing protein nuclease	*Ignisphaera aggregans*	55.8	3.00E−07
35	csu:CSUB_C0252 hypothetical protein	Candidatus *Caldiarchaeum subterraneum*	57	8.00E−07
36	thf:MA03_04400 hypothetical protein	*Thermofilum* sp. 1807-2	55.1	8.00E−07
37	mcn:Mcup_0176 nuclease	*Metallosphaera cuprina*	55.5	9.00E−07
38	iag:Igag_0323 ParB domain-containing protein nuclease	*Ignisphaera aggregans*	55.1	2.00E−06
39	ape:APE_0702.1 hypothetical protein	*Aeropyrum pernix*	52.4	5.00E−06
40	llo:LLO_2374 ParB-like nuclease	*Legionella longbeachae*	52	9.00E−06
41	mse:Msed_2111 nuclease	*Metallosphaera sedula*	52.4	1.00E−05
42	tpe:Tpen_1604 nuclease	*Thermofilum pendens*	50.8	3.00E−05
43	tcb:TCARB_0094 Y4yB	*Thermofilum carboxyditrophus*	50.1	3.00E−05
44	xbv:XBW1_4662 ParB domain protein nuclease (fragment)	*Xenorhabdus bovienii* CS03	49.7	4.00E−05
45	thb:N186_00710 hypothetical protein	*Thermofilum* sp. 1910b	49.7	6.00E−05
46	aho:Ahos_0573 ParB domain-containing protein nuclease	*Acidianus hospitalis*	49.3	2.00E−04
47	pcv:BCS7_19460 transcriptional regulator	*Pectobacterium carotovorum* subsp. odoriferum	47	4.00E−04
48	pct:PC1_3899 hypothetical protein	*Pectobacterium carotovorum* subsp. carotovorum PC	46.6	6.00E−04
49	pcc:PCC21_038910 hypothetical protein	*Pectobacterium carotovorum* subsp. carotovorum PC	46.6	6.00E−04
50	pato:GZ59_41630 hypothetical protein	*Pectobacterium atrosepticum* 21A	46.6	6.00E−04
51	patr:EV46_20445 transcriptional regulator	*Pectobacterium atrosepticum* JG10-08	46.6	6.00E−04
52	eca:ECA4109 hypothetical protein	*Pectobacterium atrosepticum* SCRI1043	46.6	6.00E−04
53	ffo:FFONT_0040 ParB-like nuclease	*Fervidicoccus fontis*	47.4	0.001

Homology search was performed by utilizing BLAST Search in GenomeNet (http://www.genome.jp/tools/blast/)

Supplementary Table 4. List of primers used for genetic analysis in this study

Use	Gene	Type	Primer name	5'-sequence-3'
For disruption vector construction	*tk1449*	A	PmetC-F	GGCCCCTCACGGCCTCCACGGCCTTTAACT
			PmetC-R	CAGAAGGCCTATCTCGGTGAGGCAGAGGTTGACGA
		B	PDmetC-F	TGCAACCACCCACGTCCACTTCCCCCACATCTTTAT
			PDmetC-R	TCATCGAGGACATAGACAGGGCCCTGGGGGGTGGTTG
	serA	A	PserA-F	AGGTCAAGAACGGCCACCTTAAGGACGATCCTCATCTCGAGGG
			PserA-R	GCAGTATAAATCCTTCCCGAAAGGTAAACGACGTCGC
		B	PDserA-F	CTCTATTTTGTCAAACTTTTTAAGTATCCTTTCTCATTTTTGATCGGTGGG
			PDserA-R	CCTTCATCCCCTCCTCTTTATTGCCATCAGCATGACCTGA
	ldhA1	A	PldhA1-F	CTCAAGGGGGTAGCCAATTGCCGCGAGGGA
			PldhA1-R	TGTCCTTTTTGGGTATCGGGACTATGACGA
		B	PDldhA1-F	TGCTGTGAGGTGGGAGCATGTGGCGGGAAG
			PDldhA1-R	GTTATCACCTTAAGATATACATCGAAACGA
	ldhA2	A	PldhA2-F	CCTTCTCAGCTATCTCCCTCGCCTTCATGT
			PldhA2-R	TGCCCTACGCCGCCAGCAAGGTCGGCCTCA
		B	PDldhA2-F	ACCCTTTGCCCCTTTCTTACATTGACCATC
			PDldhA2-R	GTCCACCACCGTTTAACAGTTTGAAAAAGG
	cysK	A	PCYSK-F	TATGCTGGAGAAAGAAAAGGAAGCCCTTGC
			PCYSK-R	ACCAAAGGTTTAGAACCCCAATTTTCACCA
		B	PDCYSK-F	ATCTTTTTTAATCTTCCCTTATCCCTTCTTGAGGG
			PDCYSK-R	CCCCCCTCTCGTCCGAAAGGGAAAGCCTGAGC
	serK	A	PserK-F	AAAGCAGAAACTCCTCATAGTTGCTGACGC
			PserK-R	ATCATGTACATCGCCCACGCCAACAACCCG
		B	PDserK-F	TGTCTCTTTCTTCCATCTTATTAACAG
			PDserK-R	TCCTGACCCTCCGTAACCTTAACCTGCAGA
	glyA	A	PglyA-F	GAGTGATAAGACGAGAGTTTTGAACCCCAA
			PglyA-R	CTCTTTCCAGCTTCCGTTTTCCAGCAGGTA
		B	PDglyA-F	ATTGGAACTTTTCCACATTTTTATTTCT
			PDglyA-R	TGGGAACACCTCGATGGTTGTTTGAACGGG
	tdh	A	Ptdh-F	GGTGGAGCTTAATTTCACCGCTTGCTACCT
			Ptdh-R	ATTACTCGATAAAGTCATGATACTTGGTGG
		B	PDtdh-F	GTGGCTTGATATTCTTCTTTCCAATTTTTC
			PDtdh-R	AGTGATCATCTCCTTAACCTTACA
For DNA sequencing of 5'- and 3'-flanking regions of target gene	*tk1449*	C	SEQDmetC-F1	CTCTCCCCCGCCGCTACCAC
			SEQDmetC-F2	GTTCAGGTTCCTGTCCTTTG
			SEQDmetC-F3	GGATGCTCTTCAAAGAGAGT
		D	SEQDmetC-R1	TTGCCAGCGATTGGTTTGAC
			SEQDmetC-R2	GACCATACTTTGGCCGGAAT
			SEQDmetC-R3	GGAAGCTTTAGCGTTTCTTC
	serA	C	SEQDserA-F1	AACTGGAAAGCTCGCAGGGT
			SEQDserA-F2	ATCGCCAGGGCGGAGCAGTT
			SEQDserA-F3	CTATCCTGGATTTTCCAATC
		D	SEQDserA-R1	GACCGAGGTTGACTACATAC
			SEQDserA-R2	TGGAGGAAGAGGTCTCAAGA
			SEQDserA-R3	TGGCTAAAAAGACAGAGGAC
	ldhA1	C	SEQDldhA1-F1	CGAAGTTCTGGCGGTAGTTG
			SEQDldhA1-F2	AACCCTGATGAGCGGCCTCG
			SEQldhA1-F3	AGGGCCGGGAAGAGAACCTC
			SEQldhA1-F4	TTTCCCGGTTTCTCCAAAAG
		D	SEQldhA1-R1	GGTTTGTGTTCTCGTGTTCA
			SEQldhA1-R2	ATCGTCAATTCCAATGTGGA
			SEQldhA1-R3	CCCCCTTGCCAGCTTCAGCA
			SEQldhA1-R4	AAGACCGTAGGGTTCGAGGA
	ldhA2	C	SEQldhA2-F1	CCCTAATGAGCTTCGGTGCG
			SEQldhA2-F2	CCTCTCGGGCCTTTGGAGCA
			SEQldhA2-F3	GGAAACCTGATACCGCTCCT
			SEQldhA2-F4	AACACAGAAAGTGAGCGCTT
		D	SEQldhA2-R1	ATTTCCCCTTCAATTTCACG
			SEQldhA2-R2	CCTCGCCGGTCAGCTTCCCT
			SEQldhA2-R3	GTCTCGGGTGAATCGTATGA
			SEQldhA2-R4	ATCAGTCCTTATAAAACTGA
	cysK	C	DCSEQ-F1	CCCGGTGCTTCAACGATAAG
			DCSEQ-F2	TACGGATGGACTACTGAGAG
			DCSEQ-F3	CTTCTATATTTCAATGGGCG
		D	DCSEQ-R1	GATAATCGAAGGAATGGAGA
			DCSEQ-R2	AGGAGCTTAGGAAGTACGAC
			DCSEQ-R3	GGGAAGAACTCCTCAAGATT
	serK	C	SEQDserK-F1	GAACAACGTCGAGGAGCGTC
			SEQDserK-F2	CAGCCACTTTAACTCCGAGT
			SEQDserK-F3	ACCTTTGCTAGGATGAGCT
			SEQDserK-F4	CAGCCTCCTCTTGGCCTCT
		D	SEQDserK-R1	CAGAACCCTGTCTCAACCGT
			SEQDserK-R2	GGGCACGTCTGGGTTGTGTT
			SEQDserK-R3	ATCCCTTTTTTGTAAGTACC
			SEQDserK-R4	GACGCTCCTCGACGTTGTTC
	glyA	C	SEQDglyA-F1	ATGAACTCCTTTCCGTTTAA
			SEQDglyA-F2	GCCGTTCTGGAGGAGCTTGC
			SEQDglyA-F3	GGGTCTTCGAGAACAGGGGA
		D	SEQDglyA-R1	GCCACTTCATCGGACTCATC
			SEQDglyA-R2	CTGAAGTGGTAGCTGGTAGT
			SEQDglyA-R3	TTTTTGAACTTCCAGAATAA
	tdh	C	SEQDtdh-F1	GAACATCGAGGTCAGTTTCA
			SEQDtdh-F2	TTGTCTATAACATCCACGAG
			SEQDtdh-F3	AACTTAGGTTTGCCGTTGTC
		D	SEQDtdh-R1	CCTCACTTAACCAGAATCCG
			SEQDtdh-R2	GTCTTCTGAGTTCCCTCCTT
			SEQDtdh-R3	CCTTTGGTAGAGAATAACAG
	universal	E	M4S	CTGGCGAAAGGGGGATGTGC
			RVS	ACACTTTATGCTTCCGGCTC
For PCR analysis of gene disruptants	*tk1449*	F	CHDmetC-F	TTTCTCTGGCCTCCCTCCAGAGGCCCAGCCATTCC
			CHDmetC-R	GACCACCTCGATGAGAAGGTCGACGAGGAGGGAA
		G	CHmetC-F	TGCGCCTCTCCTCTGGCATAGTCTTGGCGGCACTC
			CHmetC-R	AACGGTAAGGCTCCTGGAAAAGAAAGTTGCTTCTA
	serA	F	CHDserA-F	GGGAAGCTTCCCCTTCTTCGAGAGCTCCAGAATCTTCCTG
			CHDserA-R	GGGCGAAGGCGATAGTAAACTCCGTGATAAACGGTCTCGG
		G	CHserA-F	CGGGTGGTCTGCTGGAAGGGGCTCCTCCTCGAAGACATCA
			CHserA-R	AACTCGTTAAAGACGTCGATGCGATAATCGTTAGGAGCAA
	ldhA1	F	CHDldhA1-F	TCGGCTATCTCACGAGCATAGGCGAAAACG
			CHDldhA1-R	ACTCCCGCTCCCGGTAGGCCAGAAGCTCAT
		G	CHldhA1-F	GAGTTCTTGTGACATTTAAGATGAAGAGCA
			CHldhA1-R	TTCCGGCGGGCGAACCTTCACGACTTCCCT
	ldhA2	F	CHDldhA2-F	ATACTCGTTGGAACGCAGATAGGGGCTGGA
			CHDldhA2-R	TTTGAAAGCTCCTCTCTGAGCTTTGGAAGG
		G	CHldhA2-F	AGGTTTTCATAACCCGTGCCATTCCCGAGA
			CHldhA2-R	GGTTTTCTAACCTTCACGACTTCCTTATTC
	cysK	F	CHDCYSK-F	ACCTCTTTCTGCAATTGCCGAAAGACTTAT
		H	r22	CTCCGGCCTGGTCGCAGGAGCTATTACGCC
		G	CHCYSK-F	CTTTAACCCCTTCAGCAGGAGCATAAAGGA
			CHCYSK-R	TTTCTCAAACACCTCAACGTACTTAAACGC
	serK	F	CHDserK-F	AGCGAGAGCGCCCTTAAAGTGCT
			CHDserK-R	AGATGAACCTAGAGCTCCTCCAGATA
		G	CHserK-F	TCCGGAATCTTCTGGAAGCCGATGCTC
			CHserK-R	TCCTCTCTCGCGTCCTCTTTGAGGC
	glyA	F	CHDglyA-F	AAGGCCCATTCTGATCCGATAGAGAACGGC
			CHDglyA-R	CCAGTTGCCGCCCGGCGGTATCTGCCACCC
		G	CHglyA-F	GCTGAAGGATATAGAGAGTACAGAGACAAG
			CHglyA-R	CTCCCTGAGCCTGAGCGGGAGCCCGTGGTC
	tdh	F	CHDtdh-F	TATTCCGAGCTTTTTCTCAAGGGCTTTTCT
			CHDtdh-R	AGAAGCCGAATACCTAGTAACACACTACAA
		G	CHtdh-F	CGAGAAAATGCAGGCTATTATGAAGACTAA
			CHtdh-R	TCATCCCTTGTGAGGGAAGAAGACGACC

A: Primers for amplifying target genes and their 5'- and 3'-flanking regions
B: Primers for inverse PCR amplifying 5'- and 3'-flanking regions and entire plasmid to exclude target genes
C: Primers for sequencing 5'-flanking region of target genes
D: Primers for sequencing 3'-flanking region of target genes
E: Primers annealing outside of multi cloning site of pUD3 and pUD2
F: Primers annealing outside of homologous regions for homologous recombination
G: Primers annealing within the target genes
H: Primer annealing within the *pyrF* marker gene

Supplementary Table 5. List of primers used for expression vector construction in this study

Use	Gene	Type	Primer name	5'-sequence-3'
For expression vector construction	*serK*	I	TK0378-F	aaaaggggCATATGGGAGTTGAGAAGGTTCCGAAGTACG
			TK0378-R	aaaGAATTCTCAGAACAGCTCTTCGAGCTTCACGTCGAT
	tk1998		TK1998-F	aaaaggggCATATGGCGAAGACGACCTTTGAGGAGGAAAT
			TK1998-R	aaaGAATTCTCAGGCAAATGTAACGCTCCTGTCCTTGAG
For DNA sequencing of target gene	universal	J	T7P	TAATACGACTCACTATAGGG
			T7T	GCTAGTTATTGCTCAGCGG

I: Primers for amplifying target gene to be overexpressed in *E. coli* with appropriate restriction sites
Underlined sequences indicate the restriction sites.

J: Primers annealing outside of multi cloning region including NdeI and BamHI sites and used for sequencing target genes

References

1. Griffith, O. W. Mammalian sulfur amino acid metabolism: an overview. *Methods Enzymol.* **143**, 366-376 (1987).
2. Mino, K. & Ishikawa, K. A novel *O*-phospho-L-serine sulfhydrylation reaction catalyzed by *O*-acetylserine sulfhydrylase from *Aeropyrum pernix* K1. *FEBS Lett.* **551**, 133-138 (2003).
3. Westrop, G. D., Goodall, G., Mottram, J. C. & Coombs, G. H. Cysteine biosynthesis in *Trichomonas vaginalis* involves cysteine synthase utilizing *O*-phosphoserine. *J. Biol. Chem.* **281**, 25062-25075 (2006).
4. Hell, R. Molecular physiology of plant sulfur metabolism. *Planta* **202**, 138-148 (1997).
5. Kredich, N. M. & Tomkins, G. M. The enzymic synthesis of L-cysteine in *Escherichia coli* and *Salmonella typhimurium*. *J. Biol. Chem.* **241**, 4955-4965 (1966).
6. Fukunaga, R. & Yokoyama, S. Structural insights into the second step of RNA-dependent cysteine biosynthesis in archaea: crystal structure of Sep-tRNA:Cys-tRNA synthase from *Archaeoglobus fulgidus*. *J Mol Biol* **370**, 128-141 (2007).
7. Sauerwald, A. *et al.* RNA-dependent cysteine biosynthesis in archaea. *Science* **307**, 1969-1972 (2005).
8. Sato, T., Fukui, T., Atomi, H. & Imanaka, T. Improved and versatile transformation system allowing multiple genetic manipulations of the hyperthermophilic archaeon *Thermococcus kodakaraensis*. *Appl Environ Microbiol* **71**, 3889-3899 (2005).
9. Sato, T., Fukui, T., Atomi, H. & Imanaka, T. Targeted gene disruption by homologous recombination in the hyperthermophilic archaeon *Thermococcus kodakaraensis* KOD1. *J Bacteriol* **185**, 210-220 (2003).
10. Oda, Y., Mino, K., Ishikawa, K. & Ataka, M. Three-dimensional structure of a new enzyme, *O*-phosphoserine sulfhydrylase, involved in L-cysteine biosynthesis by a hyperthermophilic archaeon, *Aeropyrum pernix* K1, at 2.0A resolution. *J. Mol. Biol.* **351**, 334-344 (2005).
11. Atomi, H., Fukui, T., Kanai, T., Morikawa, M. & Imanaka, T. Description of *Thermococcus kodakaraensis* sp. nov., a well studied hyperthermophilic archaeon previously reported as *Pyrococcus* sp. KOD1. *Archaea* **1**, 263-267 (2004).
12. Yokooji, Y., Tomita, H., Atomi, H. & Imanaka, T. Pantoate kinase and

phosphopantothenate synthetase, two novel enzymes necessary for CoA biosynthesis in the Archaea. *J. Biol. Chem.* **284**, 28137-28145 (2009).
